# Isotopic tracing of glucose metabolites in human monocytes to assess changes in inflammatory conditions

**DOI:** 10.1016/j.xpro.2022.101715

**Published:** 2022-09-23

**Authors:** Ginevra Giacomello, Chotima Böttcher, Maria Kristina Parr

**Affiliations:** 1 Institute of Pharmacy, Freie Universität Berlin, Königin-Luise-Str. 2+4, 14195 Berlin, Germany; 2 Experimental and Clinical Research Center, a cooperation between the Max Delbrück Center for Molecular Medicine in the Helmholtz Association and Charité Universitätsmedizin Berlin, Berlin, Germany; 3 Max Delbrück Center for Molecular Medicine in the Helmholtz Association (MDC), Berlin, Germany; 4 Department of Neuropsychiatry and Laboratory of Molecular Psychiatry, Charité – Universitätsmedizin Berlin, corporate member of Freie Universität Berlin and Humboldt-Universität zu Berlin, Berlin, Germany

**Keywords:** Chemistry, Immunology, Mass spectrometry, Metabolism, Metabolomics

## Abstract

Differences in metabolic profiles can link to functional changes of immune cells in disease conditions. Here, we detail a protocol for the detection and quantitation of 19 metabolites in one analytical run. We provide the parameters for chromatographic separation and mass spectrometric analysis of isotopically labeled and unlabeled metabolites. We include steps for incubation and sample preparation of PBMCs and monocytes. This protocol overcomes the chromatographic challenges caused by the chelating properties of some metabolites.

## Highlights

• Protocol for the quantitation of glucose metabolites
• Protocol for the incubation and sample preparation of PBMCs and monocytes
• Chromatographic separation of cell metabolites in major metabolic pathways
• dMRM parameters for cell metabolites in major metabolic pathways

## Abstract (editor highlights)

Publisher’s note: Undertaking any experimental protocol requires adherence to local institutional guidelines for laboratory safety and ethics.

## Graphical abstract

**Figure:**
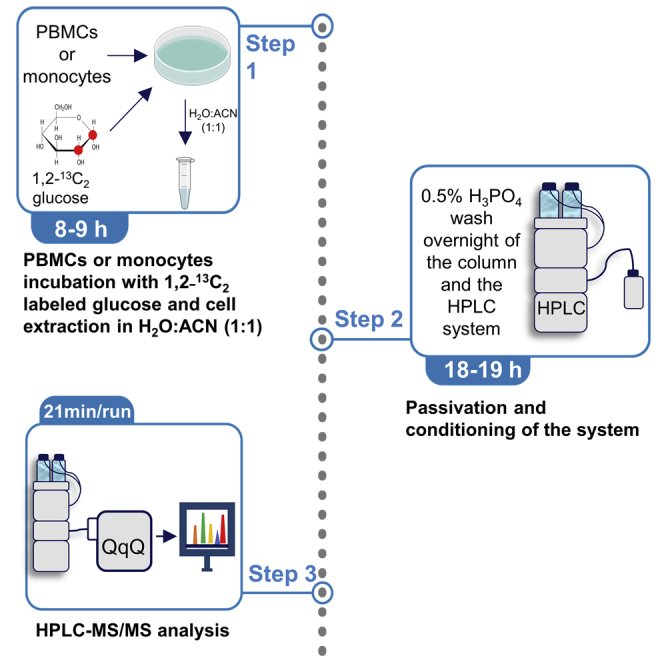

## Before you begin

### Institutional permissions

The protocol involves human immune cells and, therefore, the approval of the ethical committee is necessary before starting the analysis. The study was registered and approved by the Ethics Commission of Charité–Universitätsmedizin Berlin (Ethikkommission der Charité–Universitätsmedizin Berlin; registration number EA1/187/17), Berlin, Germany.

This protocol can be applied to the incubation of different cell types. The following paragraphs will show its application to the analysis and quantitation of the metabolites extracted from peripheral blood mononuclear cells (PBMCs) and monocytes.

Metabolic pathway(s), related metabolites, and cell type of interest should be identified before performing an experiment. It should be taken into consideration that different cell types require different culture conditions including the incubation time. For example, human cell lines are in general more stable than human primary cells (e.g., human PBMCs) and thus can commonly be longer incubated *in vitro*.

Another aspect to take into consideration is that if the analysis involves a labeled precursor, an enrichment of isotope-labeled metabolites is generally required. In this protocol, we were interested in assessing glucose metabolism in human PBMCs and monocytes (primary cells) using ^13^C-labeled glucose. Therefore, the cells had to be cultured in ^12^C-glucose-free medium supplemented with ^13^C-glucose for 4–6 h (an exact incubation time should be first validated) for an enrichment of ^13^C-labeled metabolites derived from glucose. Of note, every metabolic pathway requires a specific time to convert the precursor into related metabolites. The glycolytic intermediates are usually produced within minutes from the introduction of labeled glucose, while those of the tricarboxylic acid (TCA) cycle will need several hours (Buescher et al., 2015).

Finally, it is also challenging to distinguish metabolites that are present both in the culture medium and intracellular compartment, such as amino acids (Shlomi et al., 2014), pyruvate, or lactate (Quek et al., 2016). This continuous exchange between extra- and intracellular compartment may interfere with the incorporation of the labeled precursor and, therefore, in the determination of the metabolic fluxes.

Regarding glucose metabolism, some of the metabolites involved in the TCA cycle, glycolysis, and pentose phosphate pathway are phosphorylated substances and, therefore, are good chelating agents. The same applies to citric acid. In terms of analytics, that means a broad chromatographic peak, when even a chromatographic peak is obtainable. To overcome this issue, it is necessary to passivate the entire system with a phosphoric acid wash, as will be described in step-by-step method details. In addition, the InfinityLab deactivator (medronic acid) must be added to both mobile phase constituents. The use of alternative chelating additives to improve the peak shapes of metal-sensitive analytes has been reported, especially EDTA and other ion-pairing reagents. These alternatives, however, present some problems such as ion suppression or longer persistence in the column and the HPLC system (Hsiao et al., 2018; Pesek et al., 2011).

### ^12^C-glucose-free medium supplemented with 1,2-^13^C_2_-glucose

**Timing: 5 min**

1. Add 10% fetal bovine serum (FBS) to Dulbecco’s Modified Eagle’s Medium (DMEM), without glucose, pyruvate, glutamine, and phenol red (e.g., for the incubation of 40 million PBMCs, add 1 mL of FBS to 9 mL of DMEM).
2. Add 1,2-^13^C_2_-D-glucose in 1. to a final concentration of 4.5 g/L.

### Culture medium with unlabeled glucose

**Timing: 5 min**

3. Add 10% FBS in DMEM, without glucose, pyruvate, glutamine, and phenol red.
4. Add unlabeled glucose (^12^C-glucose) to the culture medium (3) to a final concentration of 4.5 g/L.

### Passivation solution: 0.5% phosphoric acid wash

**Timing: 10 min**

5. Add 0.5% *ortho*-phosphoric acid (H_3_PO_4_) to 90% acetonitrile (ACN) and 10% water.

To obtain 0.5 L of phosphoric acid washing solution, mix 450 mL of ACN, 50 mL of H_2_O, and 2.5 mL of H_3_PO_4_ (85%).

**CRITICAL:***Ortho*-phosphoric acid (H_3_PO_4_) 85% causes severe skin burns and serious eyes damages. Use suitable chemical protection gloves and goggles while handling it. It is also corrosive to metals, avoid contact.

**CRITICAL:** ACN is toxic by oral ingestion, dermal contact, and inhalation. It also causes eye irritation. Always use gloves, google, and lab coat and work under fume hood while handling it.

### Ammonium acetate (CH_3_COONH_4_) buffer stock solution

**Timing: 15 min**

6. Prepare a 100 mM solution of CH_3_COONH_4_ in H_2_O. To obtain 0.5 L of buffer stock solution weigh 3.85 g of CH_3_COONH_4_ and bring to volume in a 0.5 L volumetric flask.
7. Adjust pH with ammonia solution (NH_3_) to pH 9.

**CRITICAL:** NH_3_ causes severe skin burns and eye damage. Always wear gloves, google, and lab coat while handing it. It may cause respiratory irritation. Work under fume hood. It may be corrosive to metals, avoid contact. It is very toxic to aquatic life and with long lasting effects. Avoid release to the environment.

## Key resources table

**Table:** 

REAGENT or RESOURCE	SOURCE	IDENTIFIER
**Biological samples**		

PBMCs	The German Red Cross	www.drk.de

**Chemicals, peptides, and recombinant proteins**		

InfinityLab Deactivator Additive	Agilent	Cat#5191-3940
RPMI 1640 Medium	Gibco™	Cat#21875034
DMEM, no glucose, no glutamine, no phenol red	Thermo Fisher Scientific	Cat#A1443001
Acetyl-Coenzyme A Trilithium Salt BioChemica	PanReac AppliChem ITW Reagents	Cat#A3753
[1′-^13^C] Adenosine 5′-monophosphate (disodium salt)	Omicron Biochemicals, Inc.	Cat#NCT-001
Adenosine 5′-monophosphate monohydrate	Sigma-Aldrich	Cat#A2252
Adenosine 5′-triphosphate (ATP) disodium salt hydrate	Sigma-Aldrich	Cat#A1852
Citric acid	Sigma-Aldrich	Cat#251275
D-Fructose 6-phosphate disodium salt hydrate	Sigma-Aldrich	Cat#F3627
D-Glucose	Sigma-Aldrich	Cat#G7021-1KG
D-Glucose-1,2-^13^C_2_	Sigma-Aldrich	Cat#453188
DL-Glyceraldehyde 3-phosphate solution	Sigma-Aldrich	Cat#G5251
Glycine	Sigma-Aldrich	Cat#94119
Glycine (2-^13^C, 99%)	Eurisotop	Cat#CLM-136
L-Glutamic acid (1,2-^13^C_2_, 99%)	Cambridge Isotope Laboratories, Inc.	Cat#CLM-2024-PK
L-Glutamic acid hydrochloride	Sigma-Aldrich	Cat#G2128
L-Glutamine (1,2-^13^C_2_, 99%)	Cambridge Isotope Laboratories, Inc.	Cat#CLM-2001-PK
Glutamine	United States Pharmacopeia (USP) Reference Standard	Cat#1294808
Sodium L-lactate	Sigma-Aldrich	Cat#L7022
Sodium pyruvate	Sigma-Aldrich	Cat#P5280
Sodium pyruvate-2,3-^13^C_2_	Sigma-Aldrich	Cat#486191
D-(−)-3-Phosphoglyceric acid (disodium salt)	Sigma-Aldrich	Cat#P8877
D-Ribose 5-phosphate disodium salt dihydrate	Sigma-Aldrich	Cat#83875
DL-Serine	Sigma-Aldrich	Cat#68353
Acetonitrile (LC-MS grade ≥99.9%)	Fisher Scientific	Cat#326810025
Ammonium acetate (≥99%)	VWR Chemicals	Cat#84885.180
*Ortho*-phosphoric acid 85%	Merck	Cat#1.00563
NH_3_ solution 25% for LC-MS LiChropur®	Merck	Cat#5330030050
Benzonase nuclease	Sigma-Aldrich	Cat#E1014-25KU
Monensin solution (1000×)	BioLegend	Cat#420701
FBS (heat inactivated)	Gibco^TM^	Cat#10082147
LPS from E. coli O111:B4	Sigma-Aldrich	Cat#L4391-1MG
PBS (DPBS, no calcium, no magnesium)	Gibco^TM^	Cat#14200-067

**Critical commercial assays**		

MACS (Pan Monocyte Isolation Kit (human))	Miltenyi Biotec	Cat#130-096-537

**Software and algorithms**		

MassHunter 10 Quantitative Analysis program G3336	Agilent Technologies	https://www.agilent.com/
MassHunter 10 Acquisition software G3335	Agilent Technologies	https://www.agilent.com/
ChemDraw Professional 18.0	PerkinElmer	https://www.perkinelmer.com/category/chemdraw
Prism 9	GraphPad	https://www.graphpad.com/updates/prism-900-release-notes

**Other**		

1290 Infinity II LC System	Agilent Technologies	N/A
InfinityLab Poroshell 120 HILIC-Z, 2.1 × 100 mm, 2.7 μm, PEEK lined	Agilent Technologies	Cat#675775-924
1290 Infinity II in-line filter, 0.3 μm, 2 mm ID, SST	Agilent Technologies	Cat# 5067-6189
6495 QqQ with AJS-ESI source	Agilent Technologies	N/A
Water purification system LaboStar^TM^ 2-DI/-UV	LaboStar®	Cat#2206/2207
Membrane filter, non-sterile, nylon, 0.2 μm, 47 mm	Thermo Scientific	Cat#DS0215-4020
Syringe filters ROTILABO®, cellulose acetate (CA), 0,2 μm, 25 mm, sterile	Roth	Cat#KC70.1

## Materials and equipment

### LC-MS setting

For this protocol an Agilent 1290 Infinity II HPLC system was hyphenated to an Agilent 6495 QqQ mass spectrometer (MS) with an Agilent jet stream source with electrospray ionization (AJS-ESI), both controlled by MassHunter Data Acquisition software (Agilent, Waldbronn, Germany). For the separation of the metabolites, an Agilent InfinityLab Poroshell 120 HILIC-Z column (PEEK-lined, 2.1 × 100 mm, 2.7 μm) was used.

Table 1 shows the HPLC conditions and Table 2 the MS parameters. Fragmentation and source parameters were optimized using Agilent Optimizer and Agilent Source Optimizer software. The acquisition was conducted in dynamic multiple reaction monitoring (dMRM) mode in both, positive and negative mode.

**Table 1:** HPLC conditions

Autosampler temperature	4°C		
Column temperature	30°C		
Injection volume	1 μL		
Total run time	21 min		
Flow	0.3 mL/min		
Mobile phase A	10 mM CH_3_COONH_4_ (from stock solution “before you begin 6.-7.”) in H_2_O + 5 μM InfinityLab deactivator additive		
Mobile phase B	10 mM CH_3_COONH_4_ (from stock solution “before you begin 6.-7.”) in ACN + 5 μM InfinityLab deactivator additive		
Gradient	min	Solvent A (%)	Solvent B (%)
	0	10	90
	2	10	90
	12	40	60
	14	40	60
	15	10	90
	20	10	90
	Post-run (1 min)	10	90

**Table 2:** MS parameters

Agilent 6495 QqQ		
Ionization mode	Positive	Negative
Sheath gas flow (L/min)	12	12
Sheath gas temperature (°C)	350	350
Capillary voltage (V)	4500	3500
Nozzle voltage (V)	750	0
Drying gas temperature (°C)	210	210
Drying gas flow (L/min)	20	20
Nebulizer (psi)	30	30
Funnel	High P RF 190	High P RF 110
	Low P RF 40	Low P RF 60

## Step-by-step method details

This protocol can be applied to different cell cultures. Conditions of cell incubation will need previous evaluation and adjustment.

We show here, the protocols used for the incubation of PBMCs and monocytes.

Two different conditions were used in both cases: with labeled (1,2-^13^C_2_-D-glucose) and unlabeled glucose.

### PBMC incubation

**Timing: 8–9 h**

This part describes experimental steps starting with about 40 million PBMCs.

1. Thawing of PBMCs and preparation for the incubation.
  a. Warm 10 mL washing medium (10% FBS in Roswell Park Memorial Institute (RPMI) 1640 medium) in a falcon tube to 37°C in a water bath.
  b. Warm 5 mL washing medium containing benzonase (25 U/mL) at 37°C in a water bath.
  c. Thaw frozen PBMCs (max of 40 × 10^6^ cells) in a water bath (37°C). When almost completely thawed, transfer the cells under sterile condition to the falcon tube containing 10 mL washing medium (a., without benzonase).
  d. Centrifuge at 300 × *g* for 10 min at room temperature, then remove the supernatant.
  e. Gently resuspend each cell pellet in 1 mL of warmed medium with benzonase (b.), then add another 4 mL of benzonase medium. Mix well and incubate at 37°C in a water bath for 5 min.
  f. Centrifuge at 300 × *g* for 10 min at room temperature, then remove the supernatant.
2. PBMC incubation in an ultra-low attachment 6-well plate.
  a. Sterile-filter (with 0.2 μm filter) the medium supplemented with either unlabeled or 1,2-^13^C_2_-D-glucose (see the paragraph “before you begin” points 1.-2. or 3.-4).
  b. Warm the culture medium (2.a.) to 37°C in a water bath.
  c. Gently resuspend each cell pellet (1.f.) in the sterilized, warm medium (2.b.) and adjust the cell concentration to 1 × 10^6^/100 μL.
  d. Transfer about 5 × 10^6^ cells (about 500 μL) into an ultra-low attachment surface 6-well plate, add culture medium to a final volume of 1,800 μL.
  e. Incubate for 2 h at 37°C, 5% CO_2_.
  f. Add 200 μL of PBS (negative control) or 200 μL of lipopolysaccharide (LPS) solution (100 ng/mL, as a stimulant). The final volume is 2,000 μL/well.
  g. Incubate at 37°C, 5% CO_2_ for another 4 h.
3. Cell harvest.
  a. Transfer cell suspension into 2 mL Eppendorf tubes.
  b. Centrifuge at 300 × *g*, for 10 min at 4°C.
  c. Transfer the supernatant into new tubes, then centrifuge at 15,000 × *g*, for 10 min at 4°C. Take out 1 mL of supernatant and store at −80°C until measurement.
  d. Shock freeze the cell pellet in liquid N_2_ and leave it for 5 min.
  e. Take out frozen cell pellet from liquid N_2_, then add 100 μL of H_2_O:ACN (1:1). Vortex thoroughly and incubate on ice for 5 min.
  f. Centrifuge at 15,000 × *g*, for 10 min at 4°C.
  g. Carefully take 75 μL of the supernatant, without disturbing the cell pellet. Store the cell lysate at −80°C.

**CRITICAL:** All cell culture experiments should be carried out under laminar flow hood under a sterile condition.

**CRITICAL:** The use of human cells for research purposes underlies to ethical restrictions. It is necessary to obtain appropriate approvals before starting the research.

**CRITICAL:** Incubation time should be validated prior to experiment (i.e., the incubation time in 2.e. and g. can be varied and tested).

***Note:*** After isolation, PBMCs were stored in liquid N_2_ until the experiment.

***Optional:*** In step 2.f other stimulants may be applied instead of LPS.

### Monocyte incubation

**Timing: 9–10 h**

This step begins with about 40 million PBMCs.

4. Thawing of PBMCs.
  a. Warm 10 mL of medium (10% FBS in RPMI 1640 medium) in a falcon tube to 37°C in a water bath.
  b. Warm 5 mL of washing medium containing benzonase (25 U/mL) at 37°C in a water bath.
  c. Thaw frozen PBMCs (max of 40 × 10^6^ cells) in a water bath (37°C). When almost completely thawed, transfer the cells under sterile condition to the falcon tube containing 10 mL washing medium (a., without benzonase).
  d. Centrifuge at 300 × *g* for 10 min at room temperature, then remove the supernatant.
  e. Gently resuspend each cell pellet in 1 mL of warmed medium with benzonase (b.), then add another 4 mL of benzonase medium. Mix well and incubate at 37°C in a water bath for 5 min.
  f. Centrifuge at 300 × *g* for 10 min at room temperature, then remove the supernatant.
5. Separation of monocytes with the magnetic-activated cell sorting (MACS) (negative selection approach using Pan Monocyte Isolation Kit, human).
  a. Prior to MACS sorting, put the LS column at −20°C, to minimize unspecific binding.
  b. Wash the cell pellet (4.f.) with 1 mL of MACS buffer (0.5% BSA in PBS containing 2 mM EDTA) and transfer to 1.5 mL Eppendorf tubes.
  c. Centrifuge at 300 × *g*, for 10 min at 4°C, then take out the supernatant.
  d. Resuspend the cell pellet in 400 μL of MACS buffer (for 5 × 10^6^ cells).
  e. Add 100 μL of FcR blocking reagent (for 5 × 10^6^ cells).
  f. Add 100 μL of biotin-antibody cocktail (for 5 × 10^6^ cells).
  g. Mix well and incubate for 5 min in the refrigerator (2°C–8°C).
  h. Add 300 μL of MACS buffer (for 5 × 10^6^).
  i. Add 200 μL of anti-biotin micro beads (for 5 × 10^6^ cells).
  j. Mix well and incubate for 10 min in the refrigerator (2°C–8°C).
  k. Wash with 1 mL of MACS buffer.
  l. Centrifuge at 300 × *g*, for 10 min at 4°C, take out the supernatant, and then resuspend the cell pellet with 500 μL MACS buffer.
  m. Place the LS column in the magnetic field of a MACS separator.
  n. Precondition the column by rinsing with 3 mL of MACS buffer.
  o. Load the cell suspension (l.) onto the column through the pre-separation filter.
  p. Collect flow-through, which contains unlabeled cells, representing the enriched pan-monocytes.
  q. Wash column with 3 × 3 mL of MACS buffer and combine all four flow-through.
  r. Take an aliquot of 10 μL for cell count, then centrifuge the remaining cells at 300 × *g*, for 10 min at 4°C and remove supernatant.
6. Monocytes incubation in an ultra-low attachment 24-well plate.
  a. Sterile-filter (with 0.2 μm filter) the medium supplemented with either unlabeled or 1,2-^13^C_2_-D-glucose (see the paragraph “before you begin”).
  b. Warm the culture medium to 37°C in a water bath.
  c. Gently resuspend each cell pellet (5.r.) in the sterilized, warm medium (6.b.) and adjust the cell concentration to ca. 8 × 10^5^/100 μL.
  d. Transfer about 8 × 10^5^ cells (about 100 μL) of cell suspension into an ultra-low attachment surface 24˗well plate and add culture medium to a final volume of 300 μL.
  e. Add 0.3 μL of Monensin per well.
  f. Incubate for 5 h at 37°C, 5% CO_2_.
7. Cell harvest.
  a. Transfer cell suspension in 2 mL Eppendorf tubes.
  b. Centrifuge at 300 × *g*, for 10 min at 4°C.
  c. Separate the supernatant from the cell pellet (attention not to disturb cell pellet: do not aliquot the entire volume of supernatant). To analyze the culture medium, centrifuge it at 15,000 × *g*, for 10 min at 4°C before LC-MS analysis.
  d. Shock freeze the cell pellet into liquid N_2_ and leave it for 5 min.
  e. Take out from liquid N_2_ and add 100 μL of H_2_O:ACN (1:1).
  f. Vortex thoroughly and incubate on ice for 5 min.
  g. Centrifuge at 15,000 × *g*, for 10 min at 4°C.
  h. Carefully take 75 μL of the supernatant, without disturbing the cell pellet to obtain the samples of cell extract.
  i. Put the samples at −80°C or on dry ice.

**CRITICAL:** The incubation of monocytes is particularly delicate in a culture medium without pyruvate and glutamine. Verify regularly during the incubation the well-being of the cells and consider that reaching the isotopic steady state might be challenging.

**CRITICAL:** All cell culture experiments should be carried out under laminar flow box in a sterile environment.

**CRITICAL:** The use of human cells for research purposes underlies to ethical restrictions. It is necessary to obtain appropriate approvals before starting the research.

***Note:*** After collection and before the incubation, PBMCs from where monocytes were extracted, were stored at −80°C.

***Optional:*** In step 6.e different stimulants can be used, for instance, LPS to simulate different incubation conditions.

### Preparation of mobile phases

**Timing: 15 h**

8. Deactivation solution A (mobile phase A: 10 mM CH_3_COONH_4_ in H_2_O + InfinityLab deactivator additive).
  a. To obtain 1 L of mobile phase A, add 100 mL of CH_3_COONH_4_ stock solution (“before you begin”) to 900 mL of milli-Q water.
  b. Add 1 mL of InfinityLab deactivator additive per liter of mobile phase (final concentration of 5 μM).
  c. Let it rest overnight at room temperature.
  d. Filter with a 2 μm filter (non-sterile, nylon, 0.2 μm, 47 mm).
  e. Sonicate the mobile phase for 5–10 min to degas.
9. Deactivation solution B (mobile phase B: 10 mM CH_3_COONH_4_ in ACN + InfinityLab deactivator additive).
  a. To obtain 1 L of mobile phase B, add 100 mL of CH_3_COONH_4_ stock solution (“before you begin”) to 900 mL of LC-MS grade ACN.
  b. Add 1 mL of InfinityLab deactivator additive per liter of mobile phase (final concentration of 5 μM).
  c. Let it rest overnight at room temperature.
  d. Filter with a 2 μm filter (non-sterile, nylon, 0.2 μm, 47 mm).
  e. Sonicate the mobile phase for 5–10 min to degas.

**CRITICAL:** There might be some precipitation in the mobile phases, especially in the organic one (B). It is recommended to add the buffer stock solution slowly to the ACN, and only after 10–15 min the InfinityLab deactivator additive.

**CRITICAL:** ACN is toxic by oral ingestion, dermal contact, and inhalation. It also causes eye irritation. Always use gloves, google, and lab coat and work under fume hood while handling it.

### Passivation and conditioning of the system

**Timing: 18–19 h**

The passivation and conditioning of the system was conducted accordingly to Agilent’s protocol for the use of the InfinityLab deactivator (Agilent Technologies, 2018).

**Figure 1:**
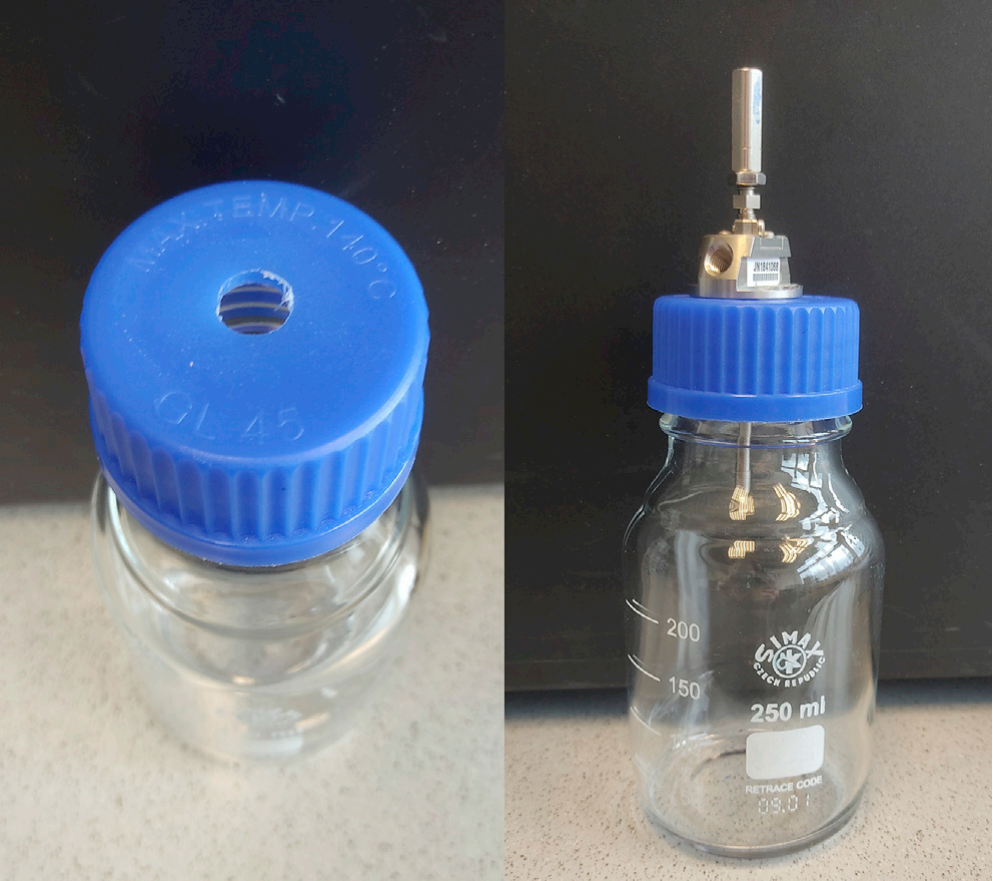
Waste container and holder for the spray needle during the passivation of the HPLC-MS system
  f. Switch the solvent in both channels to the 0.5% phosphoric acid wash (“before you begin: passivation solution: 0.5% phosphoric acid wash”).
  g. Purge the system, for 5 min at 5 mL/min with the phosphoric acid wash.
  h. Set the flow of 0.5% phosphoric acid wash to 0.1 mL/min and run for 14 h.
  i. Change the flow rate to 0 mL/min.
  j. Switch the solvent in both channels to milli-Q water.
  k. Purge the system at 5 mL/min for 10 min with milli-Q water.
  l. Set the flow of milli-Q water to 0.25 mL/min and run for 1 h through the system and the column.
  m. Change the flow rate to 0 mL/min.
  n. Switch the solvent to mobile phase A and B (“mobile phases preparation”).
  o. Purge the system with mobile phases A and B (50:50) at 5 mL/min for 5 min.
  p. Reinstall the spray needle into the MS.

10. Phosphoric acid wash.
  a. Put milli-Q water as mobile phase for both channels.
  b. Purge the system for 5 min at 5 mL/min directly to waste. If the system does not have a purge valve, momentarily detach the column, and put the inlet capillary to a waste container.
  c. Set the flow of milli-Q water to 0.25 mL/min and run for 30 min through the system and the column.
  d. Change the flow rate to 0 mL/min.
  e. Take out the spray needle from the MS source and fix it vertically in a waste container (Figure 1). Do not inject phosphoric acid wash in the MS.

**CRITICAL:** Take out the spray needle from the MS during the phosphoric acid wash. Do not inject phosphoric acid into the MS.

**CRITICAL:** During the passivation keep the spray needle in a vertical position, as shown in Figure 1, and let the sheath gas flow to prevent the formation of persistent drops of phosphoric wash along the capillary.

11. Column conditioning.
  a. Set the flow of the mobile phase to 0.2 mL/min (60% A – 40% B) and run for 30 min through the system and column.
  b. Set the flow of the mobile phase to 0.3 mL/min (60% A – 40% B) and run for 15 min through the system and column.
  c. Change the composition to 50% A – 50% B and run for 30 min through the system and column.
  d. Change the composition to 10% A – 90% B and run for at least 1 h through the system and column.

**CRITICAL:** The step-by-step increase of the percentage of mobile phase B, minimizes the risk of precipitate formation in the system.

### HPLC-MS analysis

**Timing: 21 min per run**

12. After conditioning of the analytical column, it is possible to start the analysis.

Table 3 shows the details of the dMRM method.

**CRITICAL:** Always run a couple of blanks before starting the analysis to be sure that the column is well conditioned and the pressure stable. Be aware that analysis with HILIC needs longer column conditioning.

**CRITICAL:** There might be some precipitation in the mobile phases. To our knowledge there is no suitable pre-column for both conditions of phosphoric wash and pH 9 analysis, thus the use of an in-line filter is recommended to preserve the column.

**Table 3:** dMRM method details for target analytes

Compound	Ionization	RT (min)	Quantifier (transition)	CE (eV)	Qualifier (transitions)	CE (eV)
2,3-^13^C_2_ pyruvate	[M-H]^-^	1.48	89.1 → 45.2	4		
pyruvate	[M-H]^-^	1.48	87.0 → 43.2	4		
1,2-^13^C_2_ lactate	[M-H]^-^	2.15	91.1 → 45.2	8	91.1 → 44.2 91.1 → 43.1	8 32
lactate	[M-H]^-^	2.15	89.0 → 43.2	8	89.0 → 41.1	32
2-^13^C glycine	[M+H]^+^	4.64	77.1 → 30.4	12		
glycine	[M+H]^+^	4.64	76.0 → 30.3	12		
2,3-^13^C_2_ serine^b^	[M+H]^+^	4.77	108.1 → 62.0	12	108.1→ 44.2 108.1 → 31.3	28 28
2,3-^13^C_2_ serine^a^^,^^b^	[M-H]^-^	4.77	106.1 → 75.0	8		
serine	[M+H]^+^	4.77	106.1 → 60.2	12	106.1 → 42.2 106.1 → 30.3	28 28
serine^a^	[M-H]^-^	4.77	104.0 → 74.0	8		
1,2-^13^C_2_ glutamine	[M+H]^+^	4.79	149.1 → 85.0	16	149.1 → 131.8 149.1 → 57.1	8 36
1,2-^13^C_2_ glutamine^a^	[M-H]^-^	4.79	147.1 → 128.9	8	147.1 → 42.1	36
glutamine	[M+H]^+^	4.79	147.1 → 84.0	16	147.1 → 130.0 147.1 → 56.0	8 36
glutamine^a^	[M-H]^-^	4.79	145.0 → 126.9	8	145.0 → 42.1	36
1,2-^13^C_2_ glutamic acid	[M+H]^+^	6.51	150.1 → 85.1	16	150.1 → 102.9 150.1 → 57.1 150.1 → 42.1	8 32 28
1,2-^13^C_2_ glutamic acid^a^	[M-H]^-^	6.51	148.1 → 130.0	8	148.1 → 104.0	12
glutamic acid	[M+H]^+^	6.51	148.0 → 84.0	16	148.0 → 101.9 148.0 → 56.1	8 32
glutamic acid^a^	[M-H]^-^	6.51	146.0 → 102.0	12	146.0 → 128.1	8
1-^13^C AMP	[M+H]^+^	6.83	349.1 → 135.9	16	349.1 → 118.9 349.1 → 98.0	64 32
1-^13^C AMP^a^	[M-H]^-^	6.83	347.1 → 79.0	28	347.1 → 133.9 347.1 → 97.0	36 24
AMP	[M+H]^+^	6.83	348.0 → 135.9	16	348.0 → 118.9 348.0 → 96.8	64 32
AMP^a^	[M-H]^-^	6.83	346.0 → 79.0	28	346.0 → 133.9 346.0 → 97.0	36 24
1-^13^C acetyl CoA	[M+H]^+^	7.57	811.2 → 304.1	20	811.2 → 428.1 811.2 → 158.8 811.2 → 135.8	20 64 48
acetyl CoA	[M+H]^+^	7.57	810.1 → 303.1	20	810.1 → 428.1 810.1 → 158.8 810.1 → 135.8	20 64 48
1-^13^C ribose-5-phosphate^b^	[M-H]^-^	7.53	230.1 → 96.9	16	230.1 → 138.9 230.1 → 79.0	12 40
ribose 5-phosphate	[M-H]^-^	7.53	229.0 → 96.9	16	229.0 → 138.9 229.0 → 79.0	12 40
ribose 5-phosphate^a^	[M+H]^+^	7.53	231.0 → 97.0	12		
2,3-^13^C_2_ glyceraldehyde 3-phosphate^b^	[M+H]^+^	7.67	173.0 → 99.0	20		
2,3-^13^C_2_ glyceraldehyde 3-phosphate^a^^,^^b^	[M-H]^-^	7.67	171.0 → 79.1	20		
glyceraldehyde 3-phosphate	[M+H]^+^	7.67	171.0 → 99.0	20		
glyceraldehyde 3-phosphate^a^	[M-H]^-^	7.67	169.0 → 79.1	20		
1,2-^13^C_2_ fructose 6-phosphate	[M-H]^-^	7.78	261.1 → 97.0	20	261.1 → 169.0 261.1 → 139.0 261.1 → 78.9	8 12 52
fructose 6-phosphate	[M-H]^-^	7.78	259.0 → 97	20	259.0 → 169.0 259.0 → 139.0 259.0 → 78.9	8 12 52
1,2-^13^C_2_ phosphoglyceric acid	[M+H]^+^	8.50	189.0 → 98.9	16	189.0 → 80.9 189.0 → 64.9	44 76
1,2-^13^C_2_ phosphoglyceric acid^a^	[M-H]^-^	8.50	187.0 → 78.9	40	187.0 → 96.8	12
phosphoglyceric acid	[M+H]^+^	8.50	187.0 → 98.9	16	187.0 → 80.9 187.0 → 62.9	44 76
phosphoglyceric acid^a^	[M-H]^-^	8.50	185.0 → 78.9	40	185.0 → 96.8	12
1-^13^C ATP	[M+H]^+^	8.38	509.1 → 136.0	24	509.1 → 411.0	16
1-^13^C ATP^a^	[M-H]^-^	8.38	507.1 → 158.6	32	507.1 → 409.0 507.1 → 134.0	20 40
ATP	[M+H]^+^	8.38	508.0 → 136.0	24	508.0 → 410.0	16
ATP^a^	[M-H]^-^	8.38	506.0 → 158.6	32	506.0 → 407.9 506.0 → 134.0	20 40
1,2-^13^C_2_ citric acid	[M-H]^-^	8.54	193.1 → 87.0	12	193.1 → 113.0 193.1 → 87.0 193.1 → 85.0 193.1 → 67.0	12 16 12 24
citric acid	[M-H]^-^	8.54	191.0 → 111.0	12	191.0 → 87.0 191.0 →85.0 191.0 → 67.0	16 12 24

a Transition/transitions used only for confirmation, not for quantitation.

b Transition/transitions were not experimentally confirmed due to a lack of appropriate reference material but hypothesized from the fragmentation pattern of the relative unlabeled compound.

Troubleshooting 1→ Problem 1: Precipitation in the mobile phases.

If precipitation occurs in the mobile phases (particularly in B) there will be some drops in the pressure curve of the instrument. See the protocol section “troubleshooting, problem 1” for more details.

## Expected outcomes

**Figure 2:**
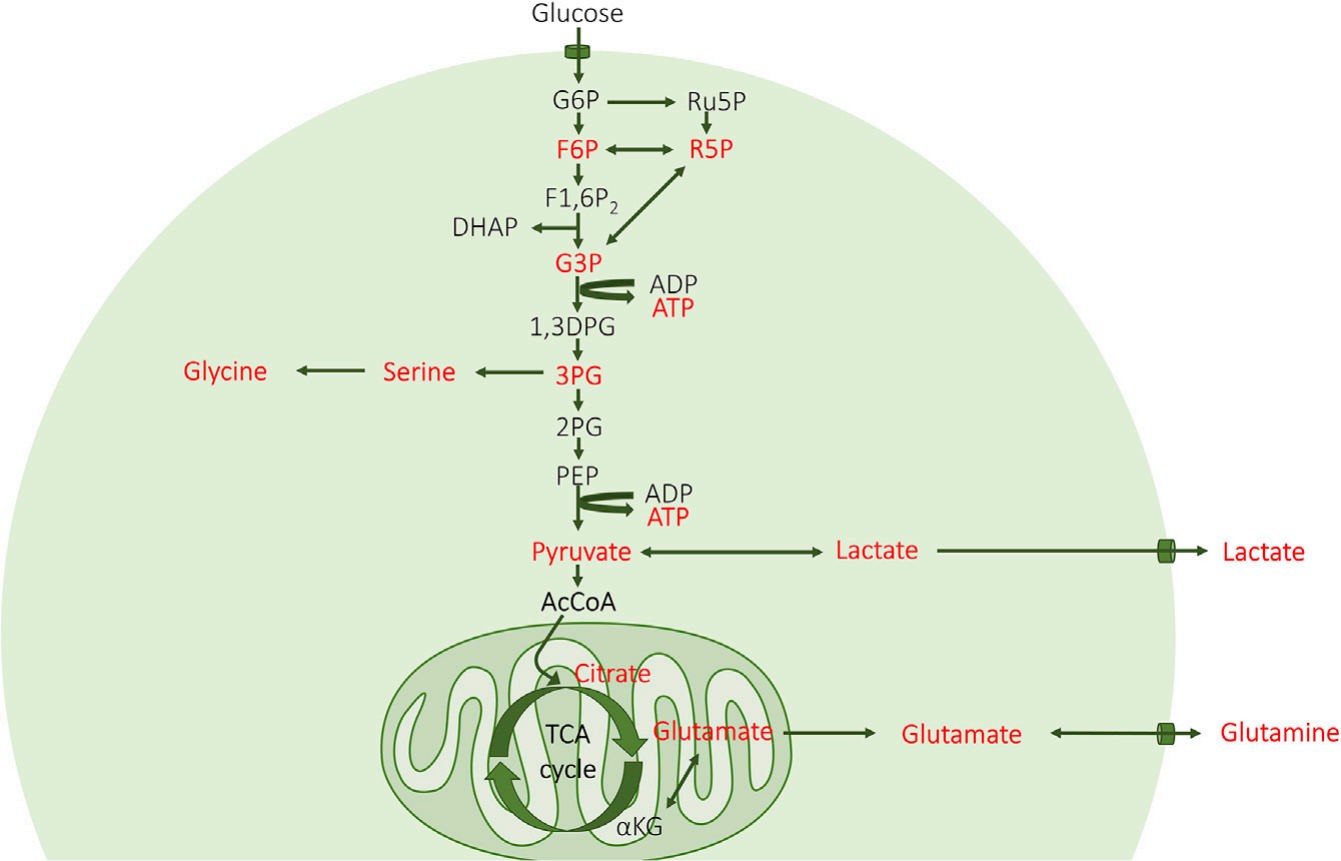
Metabolic pathways considered in this study The compounds highlighted in red were detectable and quantifiable.

**Figure 3:**
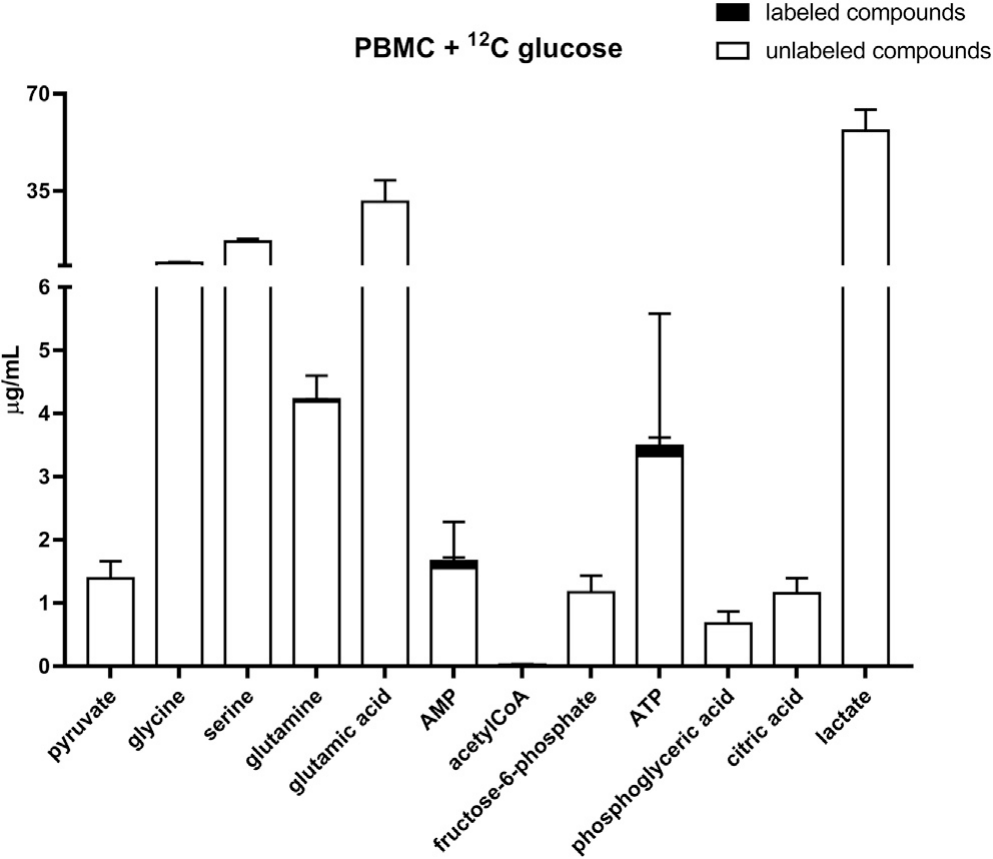
Cell extract from the incubation of PBMCs for 6 h with unlabeled glucose The white bars show the amounts of unlabeled analytes quantified in the cell extract of 5 million PBMCs. The black bars on top of the unlabeled compounds, show the detected labeled analytes. In this case only 1-^13^C AMP and 1-^13^C ATP were detected because of their masses and the natural abundance of ^13^C. The experiments were conducted in triplicates. Data are represented as mean ± SD.

**Figure 4:**
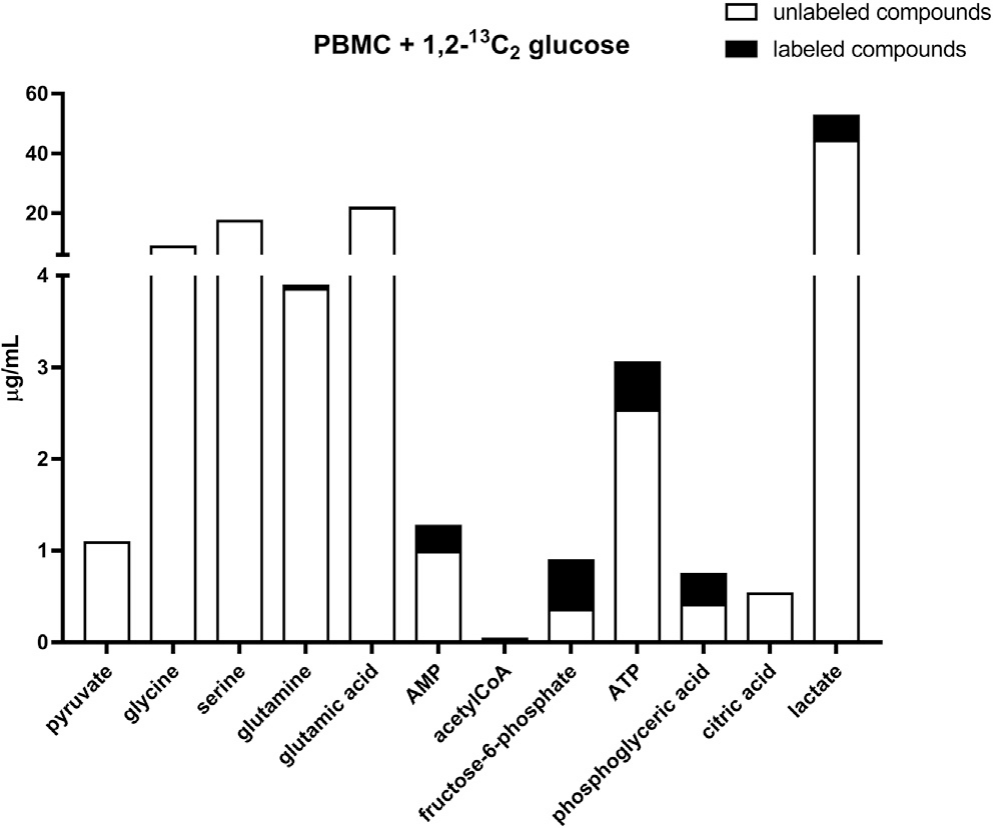
Cell extract from the incubation of PBMCs for 6 h with 1,2-^13^C_2_ labeled glucose The white bars show the amounts of unlabeled analytes quantified in the cell extract of 5 million PBMCs. The black bars on top of the unlabeled compounds, show the corresponding labeled analytes detected and quantified. The experiment was conducted in one replicate.

Data were obtained from the incubation of PBMCs and monocytes. Figure 2 shows the metabolic pathways considered, and the intermediate metabolites highlighted in red were identifiable and quantifiable in this study. The incubation of 5 million PBMCs was conducted in two different conditions: with unlabeled glucose and 1,2-^13^C_2_ labeled glucose. The amounts of the above-mentioned metabolites after incubation with glucose are shown in Figure 3 (unlabeled glucose) and Figure 4 (labeled glucose). As result, most of the labeled glucose was transformed into lactate and barely reached the TCA cycle. As discussed before, the labeled glucose may require several hours to reach the TCA cycle.

The incubation of 5 million PBMCs with unlabeled glucose (Figure 3) allowed for the detection and quantitation in the cell lysate of pyruvate, glycine, serine, glutamine, glutamic acid, AMP, 1-^13^C AMP, fructose 6-phosphate, ATP, 1-^13^C ATP, phosphoglyceric acid, citric acid, and lactate. The presence of labeled AMP and ATP is expected and justified by the natural abundance of the ^13^C isotope on earth. In fact, the higher number of carbons in ATP and AMP increases the probability of finding heavy isotope in these molecules. The incubation of 5 million PBMCs with 1,2-^13^C_2_ labeled glucose (Figure 4) allowed for the detection and quantitation in the cell lysate of pyruvate, glycine, serine, glutamine, 1,2-^13^C_2_ glutamine, glutamic acid, 1,2-^13^C_2_ glutamic acid, AMP, 1-^13^C AMP, fructose 6-phosphate, 1,2-^13^C_2_ fructose 6-phosphate, ATP, 1-^13^C ATP, phosphoglyceric acid, 1,2-^13^C_2_ phosphoglyceric acid, citric acid, lactate, and 1,2-^13^C_2_ lactate.

Monocytes (N=8 × 10^5^) were incubated in two different conditions: without a stimulation, therefore only with medium (CON) and with the addition of lipopolysaccharides (LPS). Both, cell extract and medium of the incubation, were analyzed.

**Figure 5:**
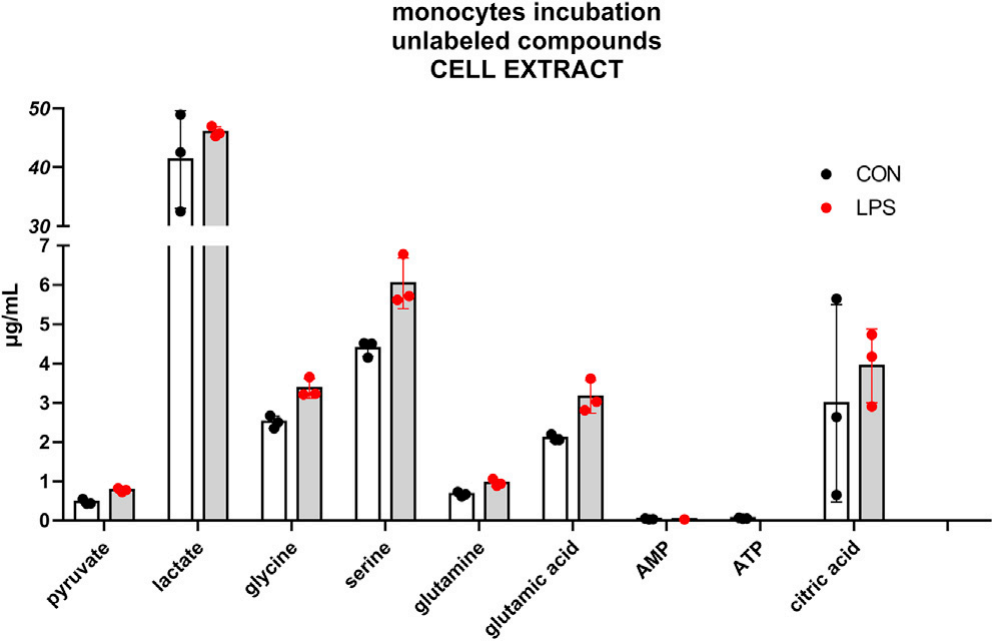
Concentration of unlabeled compounds in the cell lysate The incubation was performed with 8 × 10^5^ monocytes for 5 h with the addition of 1,2-^13^C_2_ glucose to the medium. The incubation was conducted in two different conditions: without stimulation (CON) and with the addition of LPS (LPS). The experiments were conducted in triplicates. All data points are illustrated in the graphic, and the bars represent the mean value ± SD.

**Figure 6:**
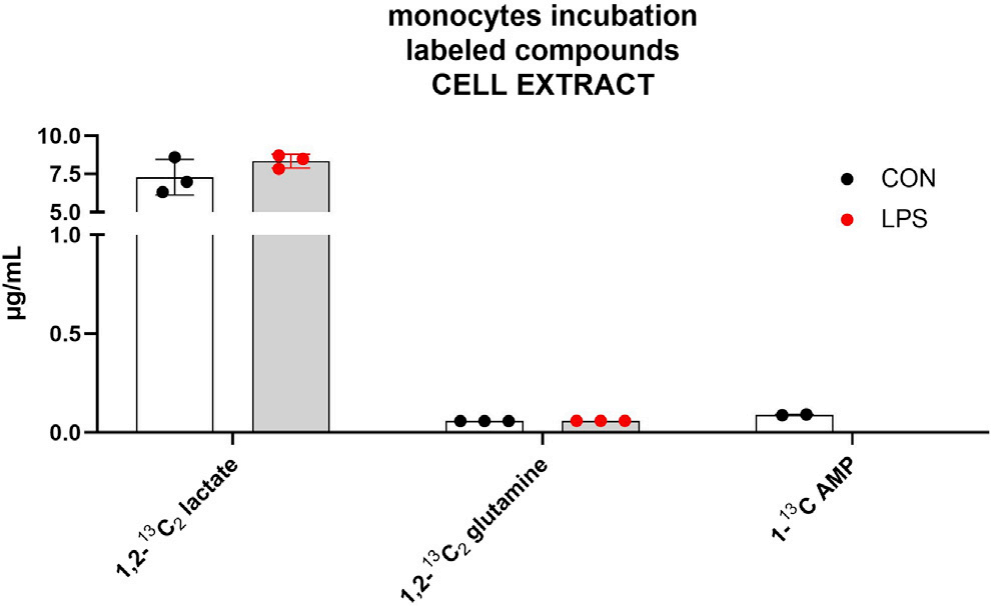
Concentration of labeled compounds in the cell lysate The incubation was performed with 8 × 10^5^ monocytes for 5 h with the addition of 1,2-^13^C_2_ glucose to the medium. The incubation was conducted in two different conditions: without stimulation (CON) and with the addition of LPS (LPS). The experiments were conducted in triplicates. All data points are illustrated in the graphic, and the bars represent the mean value ± SD.

These incubations allowed for the detection and quantitation in the cell lysate of pyruvate, glycine, serine, glutamine, 1,2-^13^C_2_ glutamine, glutamic acid, AMP, 1-^13^C AMP, ATP, lactate, 1,2-^13^C_2_ lactate, and citric acid (Figures 5 and 6 show, respectively, the unlabeled and labeled analytes).

**Figure 7:**
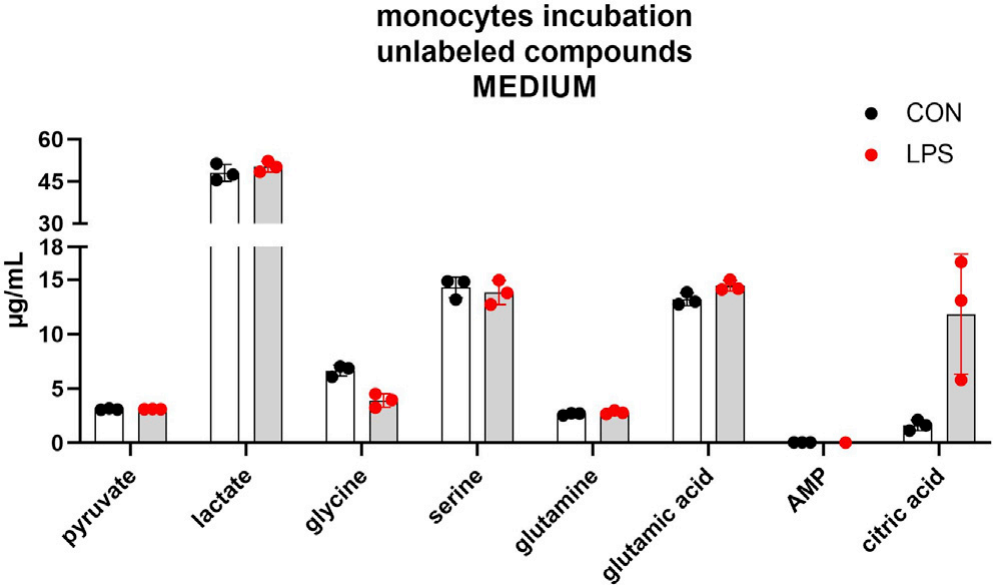
Concentration of unlabeled compounds in the incubation medium The incubation was performed with 8 × 10^5^ monocytes for 5 h with the addition of 1,2-^13^C_2_ glucose to the medium. The incubation was conducted in two different conditions: without stimulation (CON) and with the addition of LPS (LPS). The experiments were conducted in triplicates. All data points are illustrated in the graphic, and the bars represent the mean value ± SD.

**Figure 8:**
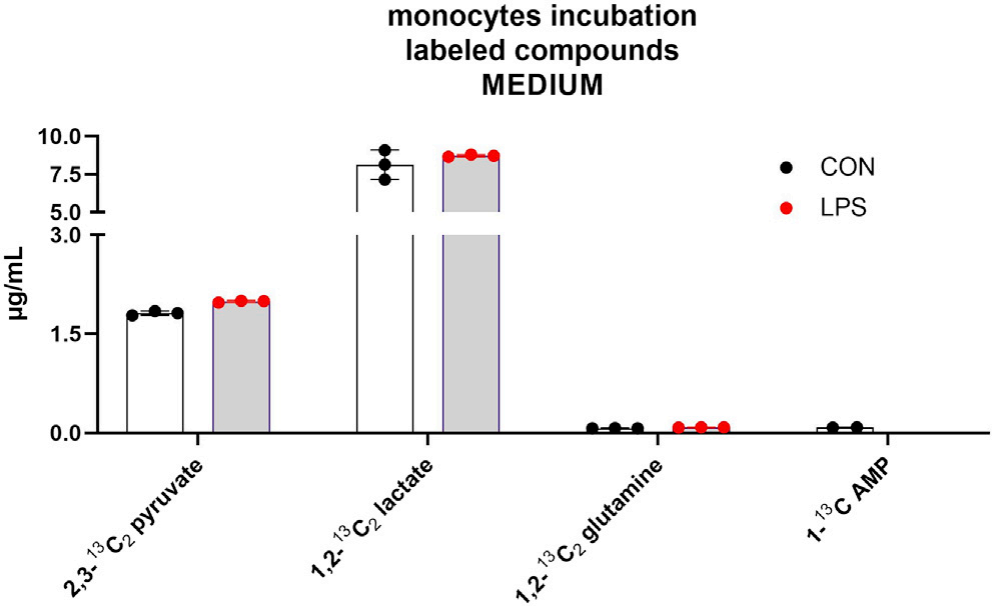
Concentrations of labeled compounds in the incubation medium The incubation was performed with 8 × 10^5^ monocytes for 5 h with the addition of 1,2-^13^C_2_ glucose to the medium. The incubation was conducted in two different conditions: without stimulation (CON), with the addition of LPS (LPS).The experiments were conducted in triplicates. All data points are illustrated in the graphic, and the bars represent the mean value ± SD.

In the medium of incubation were detected and quantified pyruvate, 2,3-^13^C_2_ pyruvate, glycine, serine, glutamine, 1,2-^13^C_2_ glutamine, glutamic acid, AMP, 1-^13^C AMP, lactate, 1,2-^13^C_2_ lactate, and citric acid (Figures 7 and 8 show, respectively, the unlabeled and labeled analytes).

The presence of lactate, glutamine, and amino acids in the incubation medium is not unexpected. On the contrary, the detection of glutamic acid, pyruvate, and citric acid is a warning sign of the well-being of the cells. These compounds cannot pass through the cell membrane, and therefore, their presence in the medium is probably due to the disruption of the membrane after the death of the cells.

In conclusion, this protocol allowed the detection and quantitation of specific compounds that are necessary to have a general overview of the well-being or the metabolic alterations of the cells. Depending on the focus of the future research and on the typology of cells used, the protocol might need adaptations. We gave an example of application to the analysis of PBMCs and monocytes, highlighting pros and cons of the method.

## Quantification and statistical analysis

The method was validated based on the ICH guideline M10 on bioanalytical method validation (EMA, 2019).

Since the matrix used is rare (PBMCs and monocytes), the validation was performed in double blanks (ACN:H_2_O), except for the matrix effect and the recovery study. Therefore, stock solutions and quality control solutions (QCs) of each standard were prepared with a concentration of 1 mg/mL in ACN:H_2_O (1:1) and stored at −80°C.

### Specificity/selectivity

The chromatographic run of 21 min and the fragmentation patterns permitted the separation of 19 compounds between labeled and unlabeled: 2,3-^13^C_2_ pyruvate, pyruvate, lactate, 2-^13^C glycine, glycine, serine, 1,2-^13^C_2_ glutamine, glutamine, 1,2-^13^C_2_ glutamic acid, glutamic acid, 1-^13^C AMP, AMP, acetyl CoA, ribose-5-phosphate, glyceraldehyde-3-phosphate, fructose-6-phosphate, phosphoglyceric acid, ATP, and citric acid.

Some of the targeted analytes, such as 1,2-^13^C_2_ lactate, 2,3-^13^C_2_ serine, 1^13^C acetylCoA, 1-^13^C ribose-5-phosphate, 2,3-^13^C_2_ glyceraldehyde, 1,2-^13^C_2_ fructose-6-phosphate, 1,2^13^C_2_ phosphoglyceric acid, 1˗^13^C ATP, and 1,2-^13^C_2_ citric acid are not commercially available to our knowledge. It is assumed, that the retention times of the labeled compounds are the same of the corresponding unlabeled ones, allowing for the identification of the targeted analyte. The suitable transitions were hypothesized based on fragmentation patterns of the unlabeled analytes. For some of them (lactate, fructose-6-phosphate, phosphoglyceric acid, ATP) the fragmentation patterns were confirmed by the results of the cell extract of PBMCs.

**Figure 9:**
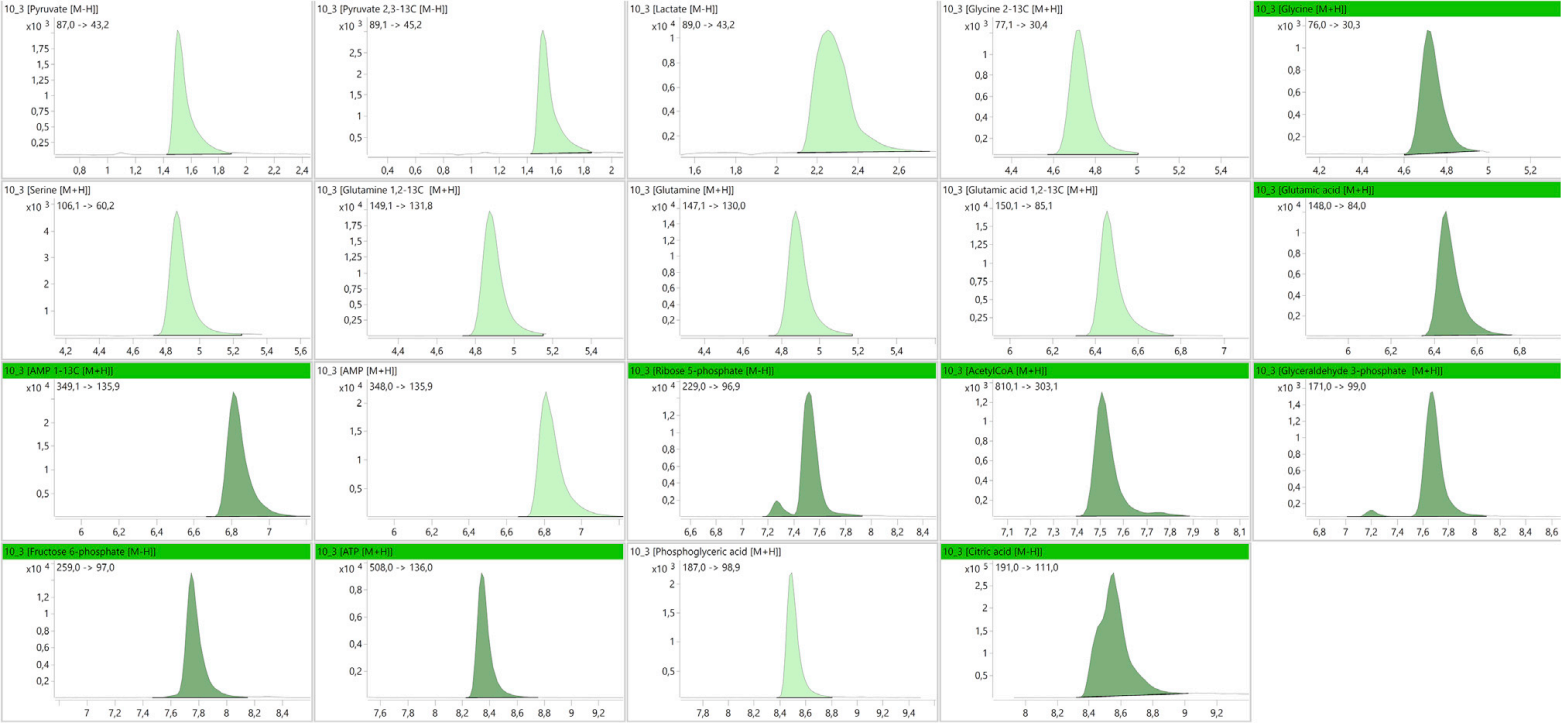
Chromatogram of a double blank spiked with standards of the targeted analytes The considered references are the following (from upper left to lower right): pyruvate, lactate, glycine, serine, glutamine, glutamic acid, AMP, acetyl CoA (acCoA), ribose-5-phosphate (R5P), glyceraldehyde-3-phosphate (G3P), fructose-6-phosphate (F6P), phosphoglyceric acid (phAc), ATP, and citric acid.

Figure 9 shows the general chromatogram of the unlabeled substances. The retention times of the relative labeled substances are virtually the same. Those analytes that could not be chromatographically separated could be distinguished by different MRM transitions as shown in Table 3.

**Figure 10:**
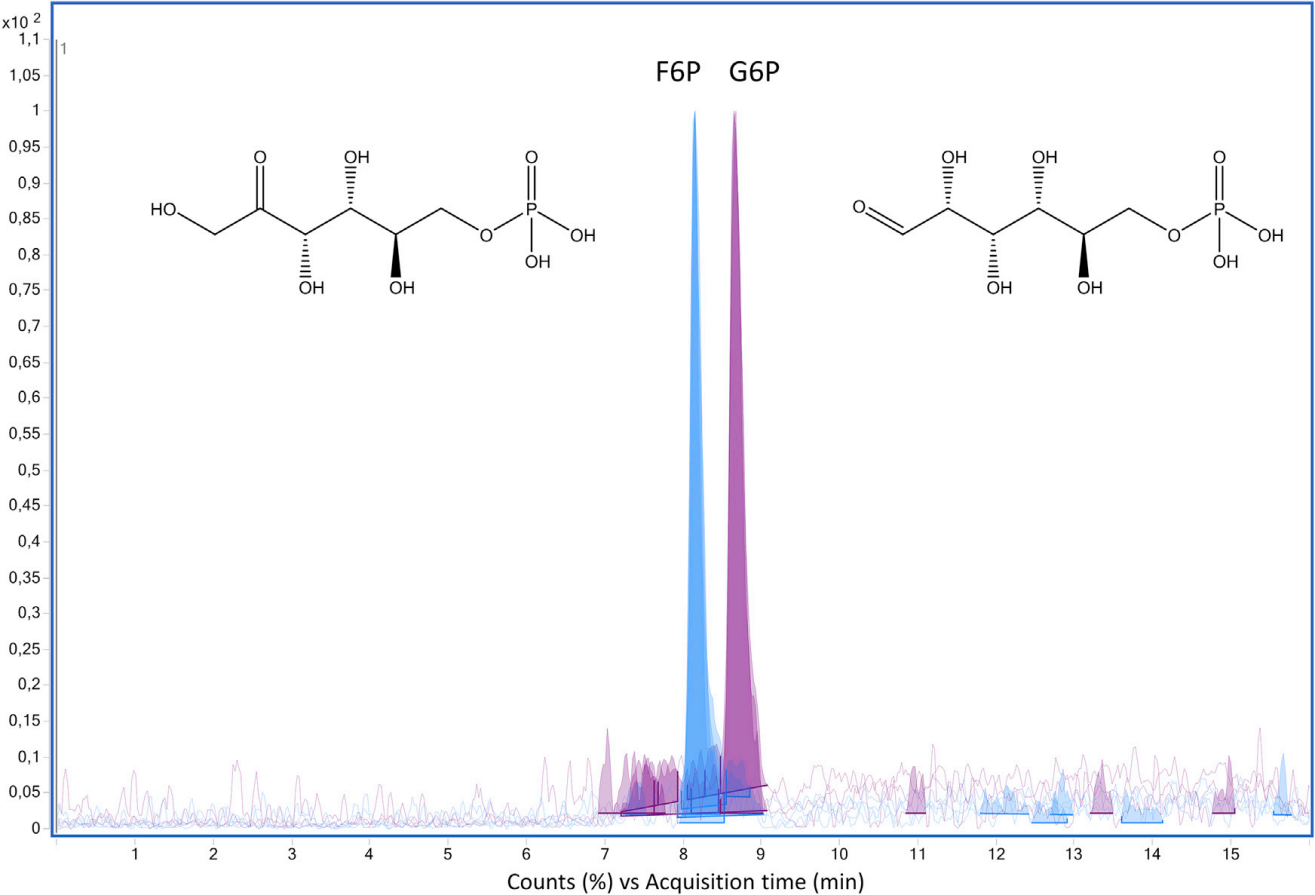
Chromatographic separation of fructose-6-phosphate (F6P) and glucose-6-phosphate (G6P)

Some compounds present in the matrix show the same molecular weight and the same ion transitions and, therefore, cause interference in the identification and quantitation. This is the case for glucose-6-phosphate and fructose-6-phosphate that have the same precursor and the same product ions and the transitions are listed in Table 3. As shown in Figure 10 though, they are chromatographically separated.

Citric acid and isocitrate have the same fragmentation pattern except for the transition m/z 191.0 → 73.0 which is characteristic for the isocitrate only. Unfortunately, they are not chromatographically separable.

**Figure 11:**
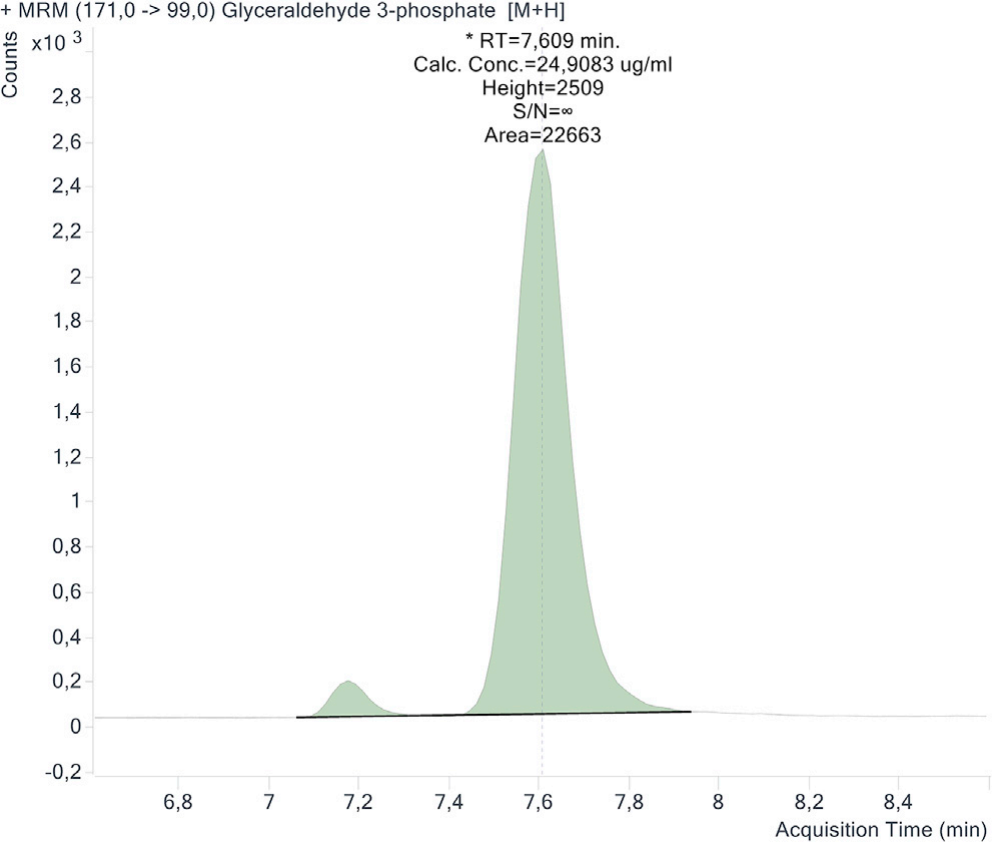
Chromatographic double peaks of glyceraldehyde-3-phosphate

**Figure 12:**
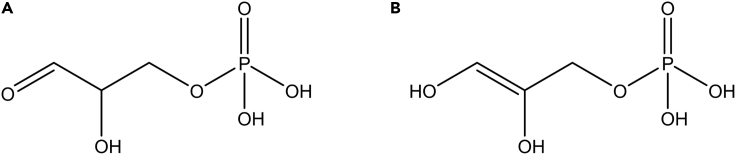
Chemical structures of glyceraldehyde-3-phosphate and its possible isomer (A) The (A) (left) shows the chemical structure of glyceraldehyde-3-phosphate. (B) The (B) (right) shows the chemical structure of the enolized form of glyceraldehyde-3-phosphate.

Glyceraldehyde-3-phosphate presents two chromatographic peaks (Figure 11), probably due to the conversion to the enolic form as shown by the structures in Figure 12.

**Figure 13:**
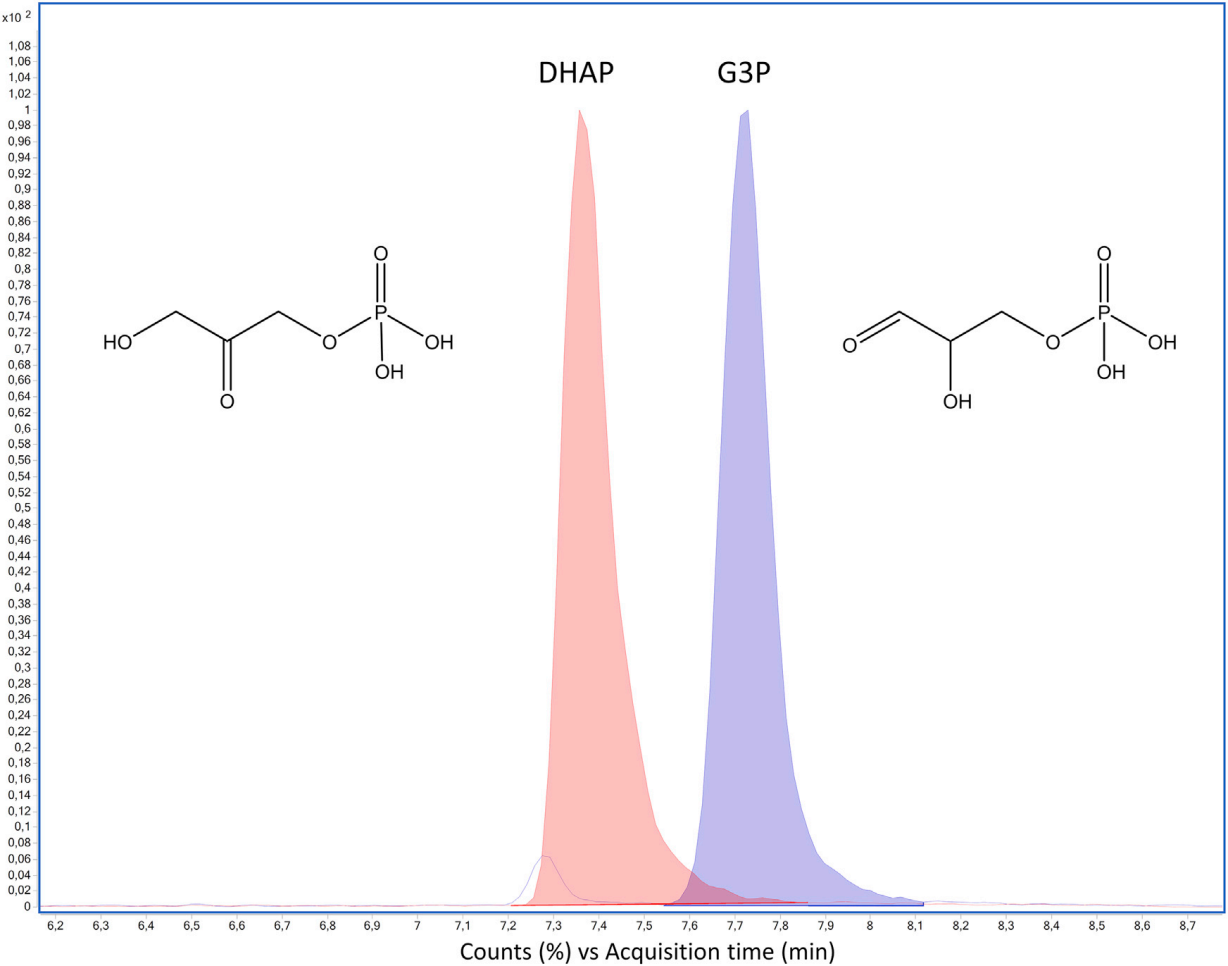
Chromatographic separation of DHAP and glyceraldehyde-3-phosphate (G3P)

Glyceraldehyde and dihydroxyacetone phosphate (DHAP) have the same molecular weight and fragmentation pattern (m/z 169 → 79.1, m/z 169 → 96.9) but they are chromatographically separated as shown in Figure 13.

### Calibration curve

For the calibration curves, at least 8 concentration levels of calibration standards were used, including lower limit quality control (LLQC), lower limit of quantitation (LOQ), middle quality control (MQC), and high quality control (HQC). The Mandel test was performed to assess the better fitting, linear or quadratic, the analysis of the variances was conducted, and the values of LOD and LOQ were calculated with the intercept of the linear regression (LOD= 3.3 × standard error intercept/slope; LOQ= 10× standard error intercept/slope). Table 4 summarizes the regression data, the LOD, and the LLOQ.

**Table 4:** Summary of the regression data, the LOD, and the LLOQ of the targeted analytes

Substance	Calibration curve range (μg/mL)	Calibration curve	Weight	Coefficient of correlation	LOD (μg/mL)	LLOQ (μg/mL)
2,3-^13^C_2_ pyruvate	0.23–50	quadratic	1/x	0.9989	0.23	0.88
pyruvate	0.18–50	quadratic	1/x	0.9947	0.18	0.3
lactate	0.42–100	quadratic	1/x	0.9980	0.42	1.29
2-^13^C glycine	0.14–50	quadratic	1/x	0.9989	0.14	0.43
glycine	0.05–50	quadratic	1/x	0.9981	0.05	0.75
serine	0.34–50	quadratic	1/x	0.9982	0.34	0.45
1,2-^13^C_2_ glutamine	0.025–50	quadratic	1/x	0.9998	0.025	0.04
glutamine	0.11	quadratic	1/x	0.9994	0.11	0.2
1,2-^13^C_2_ glutamic acid	0.076–50	quadratic	1/x	0.9996	0.076	0.1
glutamic acid	0.22–50	quadratic	1/x	0.9995	0.22	0.5
1-^13^C AMP	0.04–50	quadratic	1/x	0.9993	0.04	0.05
AMP	0.028–50	quadratic	1/x	0.9986	0.028	0.045
acetyl CoA	0.07–50	linear	1/x	0.9984	0.07	0.085
ribose-5-phosphate	1–50	linear	1/x	0.9919	1	1.9
glyceraldehyde-3-phosphate	0.1–50	Linear	1/x	0.9995	0.1	1.2
fructose-6-phosphate	0.1–50	Linear	1/x	0.9939	0.1	0.2
phosphoglyceric acid	0.05–50	Linear	1/x	0.9978	0.05	0.08
ATP	0.025–50	Quadratic	1/x	0.9983	0.025	0.077
citric acid	0.1–20	Quadratic	1/x	0.9926	0.1	0.25

### Matrix effect

In electrospray ionization, matrix effect is a confounding factor that may have a strong impact on the peak areas due to variations of ionization yield of the individual analyte.

The matrix effect was evaluated for all the target analytes in PBMCs at three different concentrations: high, medium, and different low concentrations. The analytes are endogenous compounds; therefore, their amounts were evaluated before (blank matrix) and after the spike (spiked matrix) at high, medium, and low concentrations to calculate the matrix effect. The spiked double blanks are in H_2_O:ACN (1:1).

$$matrix\;effect\;\left(\%\right)=\frac{spiked\;matrix-blank\;matrix}{spiked\;double\;blank}\times 100$$

**Figure 14:**
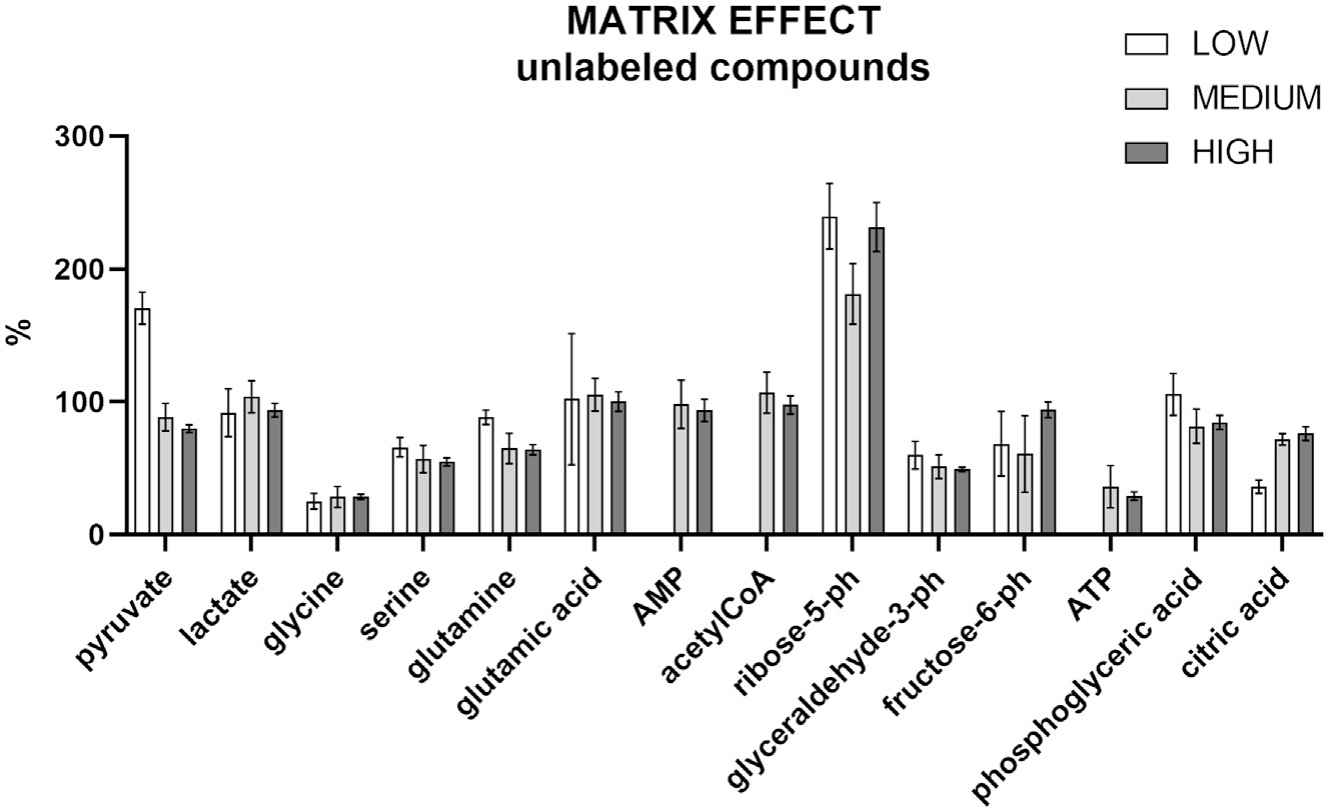
Matrix effect for the targeted unlabeled compounds The standards were spiked at three different concentrations, high (10 μg/mL), medium (5 μg/mL), and low: 0.01 μg/mL for acetyl CoA, ATP, 0.05 μg/mL for AMP, 0.1 μg/mL for glutamic acid and glutamine, 0.25 μg/mL for pyruvate, 0.5 μg/mL for citric acid, fructose-6-phosphate, glyceraldehyde-3-phosphate, glycine, phosphoglyceric acid, 1 μg/mL for ribose-5-phosphate, serine, and lactate. All measurements were conducted in sextuplicate. Data are represented as mean ± SD.

**Figure 15:**
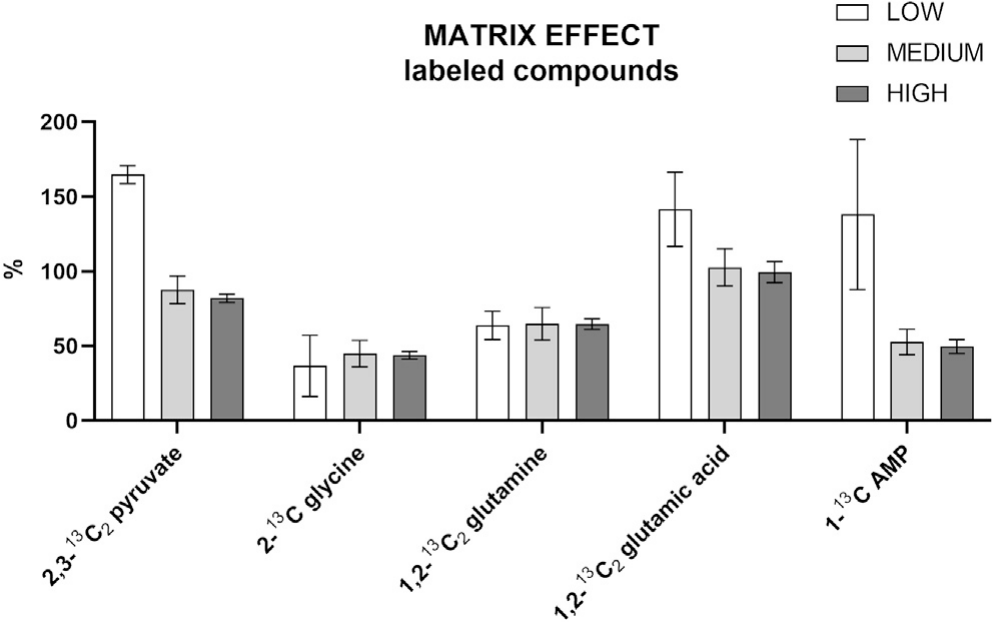
Matrix effect for the targeted labeled compounds The standards were spiked at three different concentrations, high (10 μg/mL), medium (5 μg/mL), and low: 0.01 μg/mL for 1-^13^C AMP, and 1,2-^13^C glutamic acid, 0.05 μg/mL for 1,2-^13^C_2_ glutamine, and 2˗^13^C glycine, 0.25 μg/mL 2,3-^13^C_2_ pyruvate. All measurements were conducted in sextuplicate. Data are represented as mean ± SD.

Results are shown in Figure 14 for the unlabeled compounds and in Figure 15 for the labeled compounds.

### Recovery

**Figure 16:**
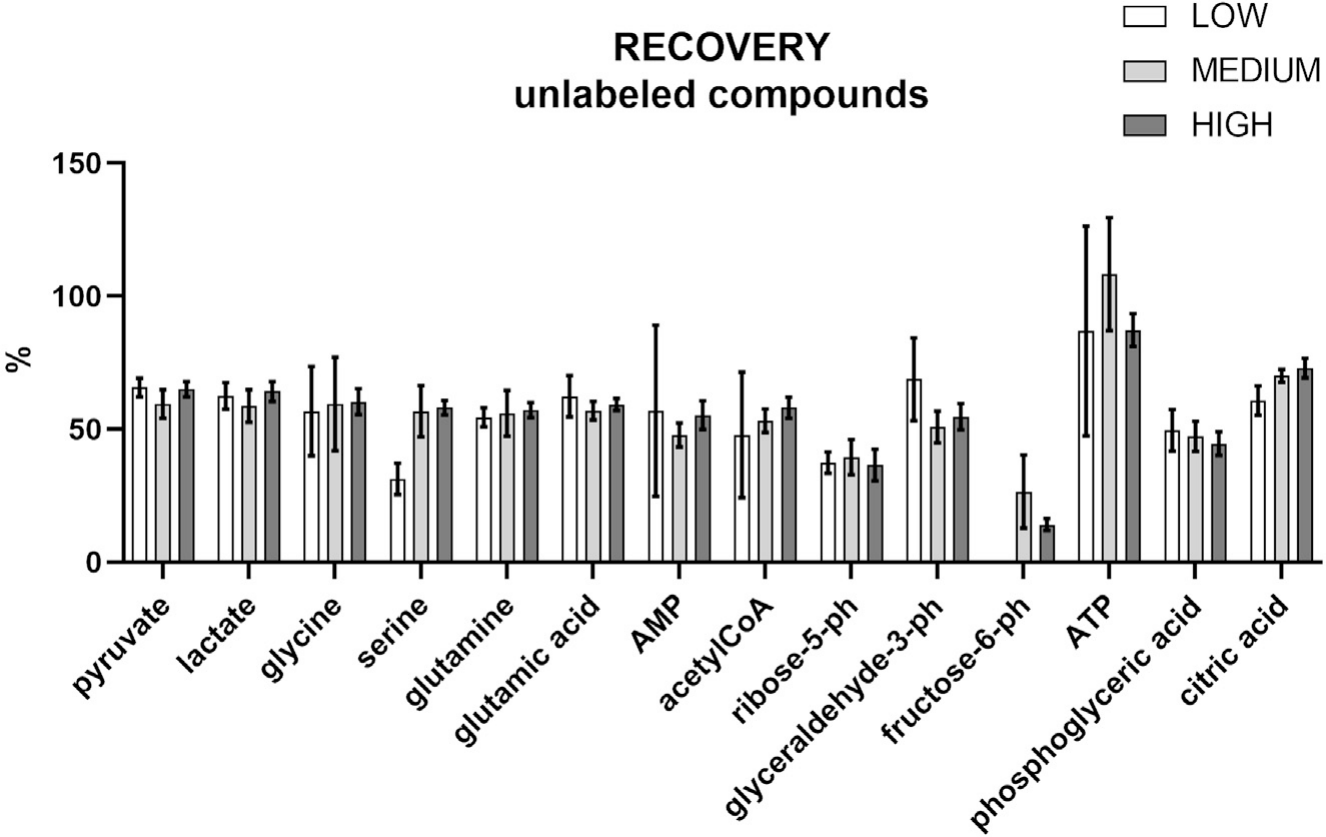
Recovery studies for the targeted unlabeled compounds The standards were spiked at three different concentrations, high (10 μg/mL), medium (5 μg/mL), and low: 0.01 μg/mL for acetyl CoA, ATP, 0.05 μg/mL for AMP, 0.1 μg/mL for glutamic acid and glutamine, 0.25 μg/mL for pyruvate, 0.5 μg/mL for citric acid, fructose-6-phosphate, glyceraldehyde-3-phosphate, glycine, phosphoglyceric acid, 1 μg/mL for ribose-5-phosphate, serine, and lactate. All measurements were conducted in sextuplicate. Data are represented as mean ± SD.

**Figure 17:**
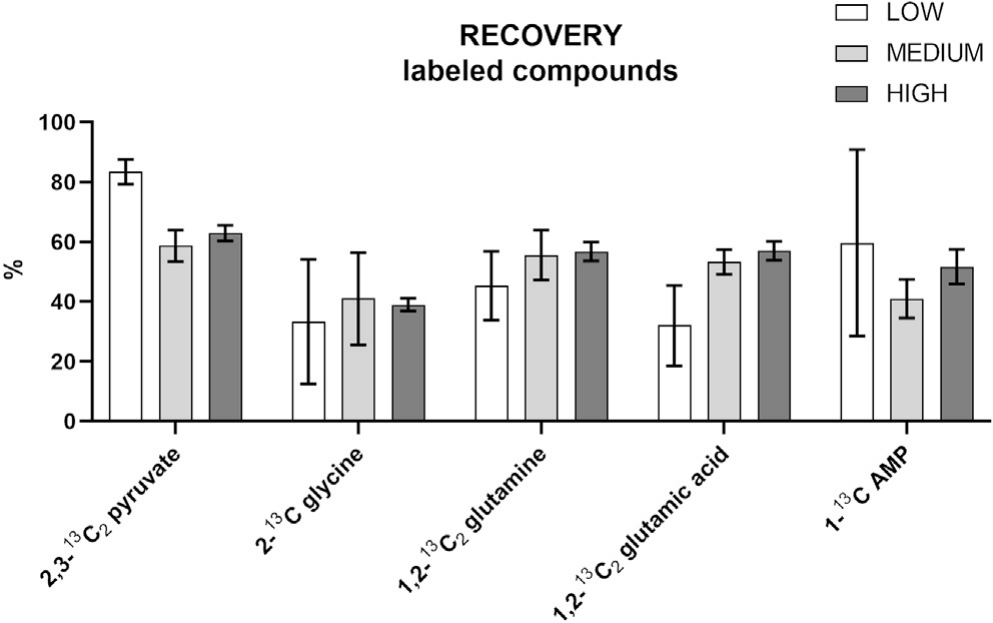
Recovery studies for the targeted labeled compounds The standards were spiked at three different concentrations, high (10 μg/mL), medium (5 μg/mL), and low: 1-^13^C AMP; 1,2-^13^C_2_ glutamic acid, 0.05 μg/mL for 1,2-^13^C_2_ glutamine, 2-^13^C glycine, 0.25 μg/mL 2,3-^13^C_2_ pyruvate. All measurements were conducted in sextuplicate. Data are represented as mean ± SD.

The recovery was evaluated for all the target analytes in PBMCs at three different concentrations: 10 μg/mL (HIGH), 5 μg/mL (MEDIUM), and different low concentrations (LOW): 0.01 μg/mL for acetyl CoA, ^13^C AMP, ATP; ^13^C glutamic acid, 0.05 μg/mL for ^13^C glutamine, ^13^C glycine, 0.1 μg/mL for glutamic acid and glutamine, 0.25 μg/mL for pyruvate and ^13^C pyruvate, 0.5 μg/mL for citric acid, fructose-6-phosphate, glyceraldehyde-3-phosphate, glycine, phosphoglyceric acid, 1 μg/mL for ribose-5-phosphate, serine, and lactate. The results obtained are shown in Figure 16 for the unlabeled compounds and in Figure 17 for the labeled compounds.

### Accuracy and precision

Intra-day and inter-day precision and accuracy were evaluated for all compounds. Four concentrations (LLQC, LQC, MQC, HQC) were injected in quintuplicates three times on the same day (intra day) and on three different days (inter-day). The results were within ±15% for CV% (precision) and ±15% for RE% for all the concentration levels. Details are reported in Table 5.

**Table 5:** Intra-day and inter-day precision and accuracy for the targeted compounds

Precision												
Substance	LLQC			LQC			MQC			HQC		
	Conc	Intra-day	Inter-day	Conc	Intra-day	Inter-day	Conc	Intra-day	Inter-day	Conc	Intra-day	Inter-day
	μg/mL	CV%	CV%	μg/mL	CV%	CV%	μg/mL	CV%	CV%	μg/mL	CV%	CV%
2,3-^13^C_2_ pyruvate	1	3.9	4.1	1.5	2.9	4.9	10	2.2	3.2	50	3.9	6.3
pyruvate	0.3	0.6	2.1	0.7	0.6	0.4	10	0.9	2	50	1.5	7
lactate	1.5	3.5	2.1	3	6.2	7.9	50	1.4	4.6	100	1.7	6.5
2-^13^C glycine	0.4	2.9	5.4	0.6	4.5	9.6	10	1.7	6.7	50	3.9	10.7
glycine	0.75	0.2	0.4	1.5	0.3	0.3	10	5.0	6.0	50	2.1	12.2
serine	0.45	0.4	0.2	2	1.0	0.8	10	1.5	4.2	50	1.5	6.8
1,2-^13^C_2_ glutamine	0.04	5.0	1.8	0.055	1.8	5.1	10	3.2	4.2	50	2.2	10.9
glutamine	0.2	2.1	2.4	0.3	1.7	3.5	10	2.9	3.3	50	3.0	11.3
1,2-^13^C_2_ glutamic acid	0.1	5.5	7.0	0.14	7.0	10.7	10	3.9	9.8	50	4.2	12.6
glutamic acid	0.5	3.5	1.7	1	2.5	2.5	10	3.5	7.0	50	4.1	14.2
1-^13^C AMP	0.05	14.9	14.5	0.18	3.2	4.4	10	3.5	5.1	50	6.1	10.2
AMP	0.045	6.9	12.7	0.065	3.0	3.3	10	3.6	11.9	50	4.4	9.6
acetyl CoA	0.085	4.5	10.6	0.45	2.6	1.1	10	3.2	6.1	50	5.8	10.2
ribose-5-phosphate	1.9	1.4	1.2	2.8	5.4	1.8	10	9.2	6.8	50	7.8	7.3
glyceraldehyde-3-phosphate	1.2	8.2	7.6	1.5	9.4	3.6	10	5.0	7.4	50	3.1	6.4
fructose-6-phosphate	0.2	1.8	1.1	0.7	3.4	0.5	10	8.4	8.5	50	5.5	6.3
phosphoglyceric acid	0.08	0.6	0.6	0.1	2.2	2.8	10	2.6	6.8	50	2.9	7.2
ATP	0.08	11	7.9	0.25	6.4	7.1	10	4.2	4.0	50	5.3	10.6
citric acid	0.25	0.3	0.6	0.8	0.3	0.3	10	4.5	3.7	50	2.6	1.6

Accuracy												
Substance	LLQC			LQC			MQC			HQC		
	Conc	Intra-day	Inter-day	Conc	Intra-day	Inter-day	Conc	Intra-day	Inter-day	Conc	Intra-day	Inter-day
	μg/mL	RE%	RE%	μg/mL	RE%	RE%	μg/mL	RE%	RE%	μg/mL	RE%	RE%
2,3-^13^C_2_ pyruvate	1	2.4	3.9	1.5	−2.4	1.2	10	2.8	−1.6	50	2.3	−0.8
pyruvate	0.3	−1.4	−0.8	0.7	0.3	0.7	10	2.8	1.7	50	−1.4	−0.6
lactate	1.5	2.2	3.1	3.0	−13.1	−6.6	50	−4.0	−5.6	100	14.2	2.2
2-^13^C glycine	0.4	−6.4	−1.1	0.6	−6.1	10.9	10	3.0	−4.2	50	8.6	−5.3
glycine	0.75	0.1	0.2	1.5	0.0	0.2	10	7.7	−1.8	50	14.9	1.7
serine	0.45	0.2	0.1	2.0	1.5	1.1	10	2.6	2.6	50	1.4	1.4
1,2-^13^C_2_ glutamine	0.04	0.1	2.6	0.055	2.9	6.5	10	5.3	5.9	50	9.1	2.1
glutamine	0.2	−3.1	−1.1	0.3	2.4	4.1	10	5.2	5.5	50	7.6	0.2
1,2-^13^C_2_ glutamic acid	0.1	−2.3	−4.6	0.14	0.9	−2.0	10	3.7	2.1	50	5.4	2.9
glutamic acid	0.5	0.6	0.2	1	2.8	1.2	10	1.3	9.5	50	2.8	1.2
1-^13^C AMP	0.05	−5	6.2	0.18	0.0	−0.1	10	−1.0	4.4	50	−0.1	−0.6
AMP	0.045	13.2	−7.7	0.065	1.5	1.0	10	−2.2	−6.3	50	−4.1	−1.9
acetyl CoA	0.085	3.3	5.8	0.45	−0.2	1.0	10	1.7	−5.1	50	−3.2	−6.2
ribose-5-phosphate	1.9	1.6	1.1	2.8	8.4	1.1	10	0.1	−9.4	50	15.6	17.6
glyceraldehyde-3-phosphate	1.2	−1.7	−9.1	1.5	−0.1	2.2	10	−11	1.2	50	−0.8	−1.6
fructose-6-phosphate	0.2	0.6	2.0	0.7	−0.7	0.1	10	0.1	−7.6	50	2.6	4.9
phosphoglyceric acid	0.08	0.2	0.1	0.1	5.7	4.9	10	7.3	3.9	50	0.5	−2.9
ATP	0.08	−1.2	0.7	0.25	−0.7	−0.7	10	2.5	−1.9	50	15	8.0
citric acid	0.25	0.2	0.6	0.8	0.5	0.6	10	8.4	3.7	50	−0.4	1.2

### Carry-over

The analyzed compounds did not show carry-over.

### Stability

The stability of the analytes was evaluated in triplicate and different conditions: at room temperature (0 h, 4 h, 8 h), at 4°C (0 h, 8 h, 24 h, 48 h, 168 h), and at −80°C (1 week, 4 weeks, 6 months, 1 year). Moreover, the freeze-thaw stability was evaluated after three cycles. The obtained results are all within ±15%.

## Limitations

A limitation of this method is the number of cells that are necessary to obtain a sufficient concentration of compounds to analyze and quantify. Therefore, is not recommended to apply this protocol to experiment sets with a very limited number of cells.

The concentrations taken into consideration in this protocol are wide, and there is a high variability of concentrations in the cell extract. For instance, the concentrations of lactate are clearly in a different range in comparison with ATP or AMP.

This protocol describes an *in vitro* study; therefore, it is a model that might differ from the metabolism *in vivo*. This aspect must be taken into consideration while evaluating the data.

## Troubleshooting

### Problem 1

Precipitation in the mobile phases.

**Figure 18:**
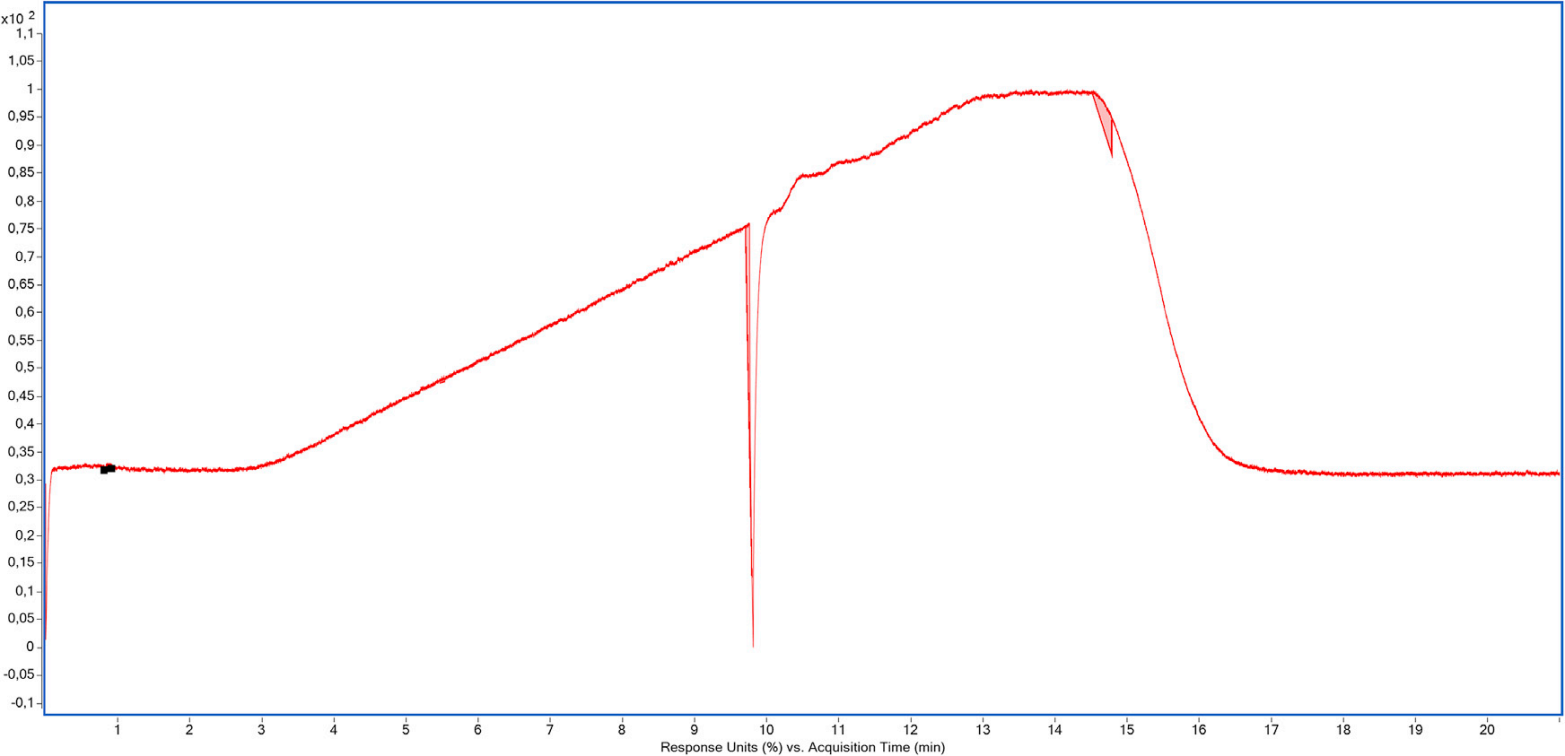
Troubleshooting due to the formation of precipitate in the mobile phases: The pressure line of the pump shows a sudden decrease

After some days, if the temperature of the laboratory is not highly controlled, the formation of precipitate in the mobile phases may be observed. As a result, the pressure of the system will show a general increase and some quick and temporary pressure drops as shown in Figure 18. After a while, these drops will become more frequent and longer.

Since there is not a pre-column capable to resist both pH, acidic for the passivation and basic for the analysis, the introduction of an in-line filter with a pore size of 0.2 μm or 0.3 μm is recommended to prevent the possible occlusion of the column due to precipitation in the mobile phases.

### Potential solution

The best way to solve these pressure drops is to clean the system with pure water for 15–30 min and redo the passivation of the system afterwards.

### Problem 2

Citric acid peak is difficult to integrate.

### Potential solution

It is extremely important to use as much as possible steel-free capillaries, column fittings, and connectors. Another possible solution, not yet tested, is the use of a bioinert system.

### Problem 3

Some cells, like monocytes, show some vulnerability during the incubation in a medium without glutamine and pyruvate and start to die already after 4–5 h.

### Potential solution

Do not plan very long incubation of these vulnerable cells or optimize the incubation conditions in advance. Remember also that the incorporation of labeled glucose into the TCA cycle takes a longer time, and the use of labeled glutamine instead might be considered.

### Problem 4

Some chromatographic peaks, like citric acid, might need manual integration to obtain the correct quantitation.

### Potential solution

Carefully review every data and integrate manually, if necessary. Be consistent, in calibration curves and samples, to obtain a correct quantitation.

### Problem 5

If PBMCs are obtained from blood donations, they might not be checked for infectious diseases.

### Potential solution

Be sure to handle the samples with the right laboratory equipment: always wear gloves, lab coat, and googles.

## Resource availability

### Lead contact

Further information and requests for resources and reagents should be directed to and will be fulfilled by the lead contact, Maria K. Parr (maria.parr@fu-berlin.de).

### Materials availability

This study did not generate new unique reagents*.*

## Data Availability

This study did not generate original code.
